# Intermittent Fasting and Fat-Free Mass Outcomes in Middle-Aged and Older Adults: A Scoping Review

**DOI:** 10.1016/j.advnut.2026.100663

**Published:** 2026-07-02

**Authors:** Sara Santero, Bojana D Blagojević, Mohammed Merzah, José Antonio Celada Guerrero, Hellas Cena, José Maria Ordovas, Lidia Daimiel Ruiz

**Affiliations:** 1 Laboratory of Dietetics and Clinical Nutrition, Department of Public Health, Experimental and Forensic Medicine, University of Pavia, Pavia, Italy; 2 University of Novi Sad, Faculty of Agriculture, From Farm to Pharm Group, Nutriomics Lab, Novi Sad, Serbia; 3 Department of Public Health and Epidemiology, Faculty of Medicine, University of Debrecen, Debrecen, Hungary; 4 Department of Community Health Techniques, Polytechnic College - Karbala, Al-Furat Al-Awsat Technical University, Kufa, Iraq; 5 Nutritional Control of the Epigenome Group, Precision Nutrition and Obesity Program, IMDEA Nutrition, UAM + CSIC, Madrid, Spain; 6 Istituti Clinici Scientifici Maugeri IRCCS, Unit of Clinical Nutrition, Pavia, Italy; 7 Nutrition and Genomics Laboratory, Jean Mayer USDA Human Nutrition Research Center on Aging, Tufts University, Boston, MA, United States; 8 Centro de Investigación Biomédica en Red Fisiopatología de la Obesidad y la Nutrición, Institute of Health Carlos III, Madrid, Spain

**Keywords:** fasting, time-restricted eating, alternate-day fasting, intermittent fasting, muscle mass, fat-free mass, body composition, older adults, middle-aged adults

## Abstract

Intermittent fasting (IF) has gained popularity as a weight-loss strategy. Because muscle mass naturally declines with age, concerns arise about effects on fat-free mass (FFM) in aging populations. The objective of this review was to explore the current evidence on the effects of IF on: *1*) FFM in middle-aged and older adults, compared with habitual diet (HD) or continuous energy restriction (CER); and *2*) other anthropometric outcomes, functional performance, and quality of life. Three databases were searched, supplemented with snowballing and the clinicaltrials.gov registry. Eligible studies (2000–2025) were randomized controlled trial (RCTs) in adults ≥45 y, with IF lasting ≥4 wk, reporting FFM using validated methods, and excluding participants with disease-related muscle loss. Twenty RCTs (1653 participants; 64% females), primarily involving adults with overweight/obesity, were included. Over the intervention durations studied (commonly ≤12 wk), between-group differences in total FFM were generally small. Findings showed that IF produced reductions in fat mass (FM), body weight (BW), and waist circumference that were comparable with those observed with CER. Reductions in BW and FM were more consistently reported in IF compared with HD. Alternate-day fasting and alternate-day modified fasting were commonly studied and frequently associated with BW and FM reductions, particularly compared with HD. Evidence regarding insulin-related outcomes, dietary protein intake, and combined IF and physical activity interventions was limited and heterogeneous. Functional and quality of life outcomes were rarely assessed. Most studies measured total FFM or lean mass using dual-energy X-ray absorptiometry or bioelectrical impedance analysis; thus, it may not reflect skeletal muscle changes. Collectively, the current evidence does not permit definitive conclusions regarding the impact of IF on FFM preservation, especially in the long-term, in older populations. The findings highlight substantial gaps in study duration and methodological standardization. Longer-term, high-quality trials incorporating standardized muscle health and functional outcomes are needed to clarify the role of IF in strategies for healthy aging.

## Graphical abstract

**Figure:**
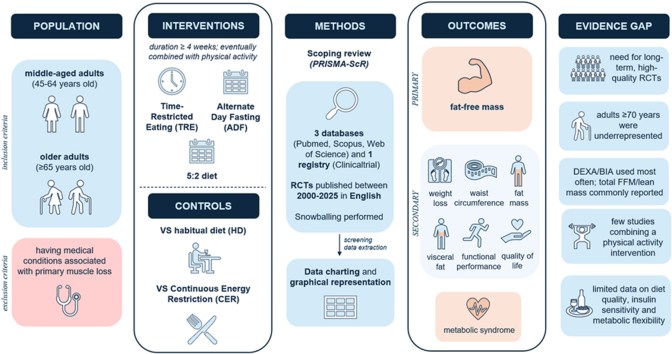

**Statement of Significance**

Intermittent fasting is increasingly studied as a dietary strategy for weight management and metabolic health, but its effects on body composition in aging populations remain incompletely characterized. This scoping review maps randomized trial evidence on intermittent fasting and FFM-related outcomes in middle-aged and older adults, including the methods used to assess body composition, the fasting regimens tested, comparator groups, PA components, and the availability of functional or patient-centered outcomes. The review highlights important evidence gaps, particularly the limited use of appendicular lean mass, muscle strength, physical performance, sarcopenia-related endpoints, long-term follow-up, and analyses focused on adults aged ≥70 y.

## Introduction

During the last two decades, intermittent fasting (IF) regimens have emerged worldwide as widely adopted dietary strategies in adults, particularly for weight-loss purposes [1]. The rapidly growing publication trends on these topics have led to the need for a shared fasting terminology, which was established in the 2024 international consensus [2]. Fasting is defined as voluntary abstinence from some or all foods, or foods and beverages, for preventive, therapeutic, religious, cultural, or other reasons. The term modified fasting is used when energy intake on fasting days is limited to up to ∼25% of energy needs [2].

On the basis of duration, fasting can be categorized into short-term fasting (2–3 d), prolonged fasting (≥4 consecutive days), and periodic fasting. The latter includes repeated fasting at regular intervals (e.g., daily, weekly, or monthly) and encompasses IF regimens [2].

**FIGURE 1:**
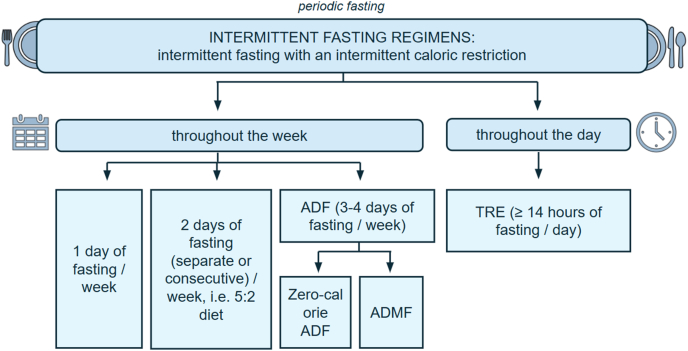
Different types of intermittent fasting regimens. ADF, alternate-day fasting; ADMF, alternate-day modified fasting; TRE, time-restricted eating.

IF interventions (Figure 1) involve intermittent caloric restriction, alternating periods of fasting or modified fasting with periods of feeding throughout the week or day. IF refers to repetitive fasting periods lasting ≤48 h each, including regimens such as 1 fasting day per week, 2 separate or consecutive fasting days per week (5:2 diet), alternate-day fasting (ADF), alternate-day modified fasting (ADMF), and time-restricted eating (TRE) [2]. Specifically, ADF alternates a day of ad libitum eating with a day of water-only fasting, whereas ADMF alternates an ad libitum day with a day of modified fasting (i.e., starting fast after a low-calorie meal, usually breakfast), aiming for better adherence of participants. TRE restricts food and caloric beverage intake to a defined window during the day, resulting in a daily fasting period of ≥14 h [2]. Notably, most IF regimens prescribe caloric restriction, except for TRE, which does not explicitly restrict caloric intake; nevertheless, participants commonly reduce energy intake due to the shortened eating window [1,3].

Regarding the benefits of IF, two recent umbrella reviews [4,5] have highlighted favorable effects—especially for ADF and ADMF—on anthropometric and cardiometabolic outcomes in adults. ADF and the 5:2 diet typically induce similar weight loss (4%–8%) over 8 to 12 wk in adults with obesity, whereas TRE produces more modest reductions (3%–4%) [3]. When comparing IF regimens with caloric energy restriction (CER), evidence consistently suggests comparable efficacy for body weight (BW) and anthropometric improvements [[6], [7], [8], [9], [10]], with even greater benefits when IF is combined with physical activity (PA) [[11], [12], [13]].

Focusing on body composition, most studies have reported a similar ratio of fat mass (FM) to fat-free mass (FFM) loss (∼75%–25%) in IF and CER interventions among adults with obesity [3,14]. Compared with habitual diet (HD), IF leads to significant reductions in BW, BMI, and FM, but generally not in FFM, in adults ≥40 y with overweight or obesity without metabolic disease [15]. In healthy adults (≥18 y), IF interventions produce comparable anthropometric improvements, with slightly greater reductions in FFM in some trials [16].

Despite promising evidence and rapid population aging worldwide [17], no clear consensus exists on the impact of fasting on muscle mass in middle-aged and older adults. This uncertainty raises concerns regarding the suitability and safety of IF in populations already prone to age-related muscle loss [3]. Physiologically, aging is characterized by a gradual decline in muscle mass and a more rapid decline in muscle strength [18]. Because low muscle mass is strongly linked to frailty, reduced mobility, and loss of independence, its preservation is a public health priority, reflected in diagnostic criteria for both malnutrition [19] and sarcopenia [20,21].

Thus, the aim of this scoping review is to clarify the impact of fasting—with or without caloric restriction and with or without combined PA—on FFM (kg) in middle-aged and older adults. Secondary outcomes include FM (kg), visceral fat (kg), weight loss (kg or %), waist circumference (WC; cm), quality of life (QoL), and functional performance.

This review mapped and synthesized the available evidence regarding the effects of fasting interventions on FFM in middle-aged and older adults, compared with no dietary intervention or alternative dietary strategies. Particular attention was given to the direction and magnitude of reported changes in FFM across studies. Additionally, the review examined evidence on the combined implementation of fasting and PA interventions, describing observed patterns of change in FFM and identifying areas where evidence remains limited or inconsistent.

Given the heterogeneity of fasting regimens, measurement methods, and populations [including adults with and without metabolic syndrome (MS)], a scoping review represents the most appropriate methodological approach. This design allows for comprehensive mapping of diverse evidence, identification of conceptual ambiguities, and synthesis of findings across regimens and populations. Because aging is accompanied by anabolic resistance, changes in protein metabolism, and higher risk of sarcopenia, understanding whether IF can be safely implemented in older adults has major clinical and public health relevance. Clarifying this issue is essential to balance the potential metabolic benefits of fasting against the risks of skeletal muscle mass loss up to the development of sarcopenia with its unfavorable associated health outcomes (e.g., reduced QoL, a higher risk of falls and fractures, and a higher risk of mortality) [22].

**FIGURE 2:**
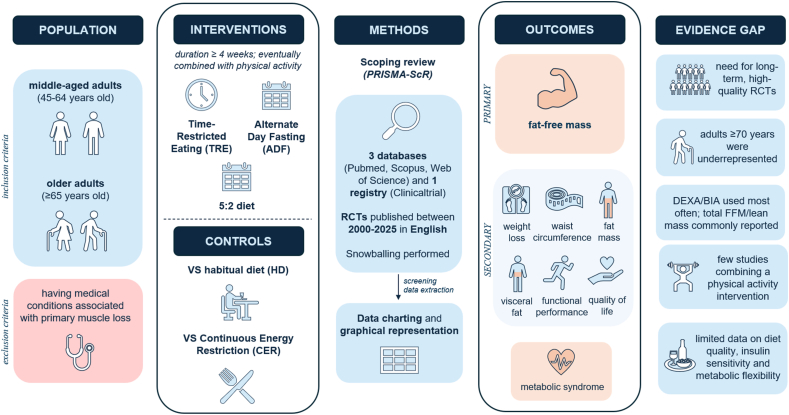
Schematic overview of the scoping review. ADF, alternate-day fasting; BIA, bioelectrical impedance analysis; CER, caloric energy restriction; DEXA, dual-energy X-ray absorptiometry; FFM, fat-free mass; HD, habitual diet; RCT, randomized controlled trials; ScR, Scoping Reviews; TRE, time-restricted eating.

The overview of our research question and performed work is schematically presented in Figure 2.

## Methods

### Protocol and registration

This scoping review was conducted following the PRISMA extension for Scoping Reviews (PRISMA-ScR) guidelines [23]. The PRISMA-ScR checklist is provided in the Supplementary Materials. The final protocol was registered on the Open Science Framework (OSF) in August 2025 [24].

### Information sources and search strategy

The research question guiding this review was “*What is known from the literature about the impact of fasting, with or without caloric restriction and with or without a physical activity intervention, on fat-free mass preservation in aging adults and older adults compared with no intervention or other dietary interventions?*”

Three databases—PubMed, Scopus, and Web of Science—were searched. A combination of keywords and Boolean operators (AND, OR) was developed through pilot searches, initially outlined by two reviewers and refined through team discussion. Detailed search strategies for each database are available in the OSF protocol [24].

Additional articles were identified through snowballing, reference list searches, and screening of registered trials on clinicaltrials.gov [25]. Only human studies published in English between 1 January, 2000 and 15 February, 2025 were included. This time frame was chosen based on publication trends showing rapid growth in fasting-related research among aging adults from 2000 onward. Preprints were excluded; however, ongoing registered trials without results were identified and summarized descriptively. Grey literature was not searched.

### Eligibility criteria and study selection

**FIGURE 3:**
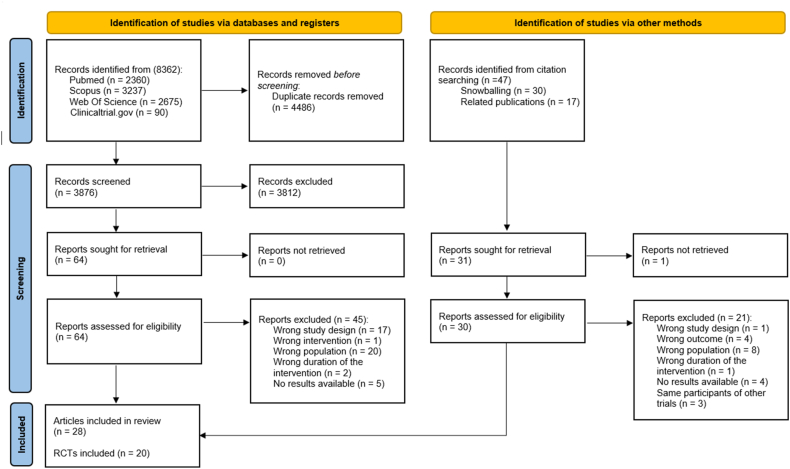
PRISMA flowchart for study selection. Source: Paje M et al. BMJ 2021;372n71. Doi 10.1136/bmj.n71. This work is license under CC BY 4.0. To view a copy of this license, visit https://creativecommons.org/licenses/by/4.0/. RCT, randomized controlled trials.

The study selection process is summarized in Figure 3. After export of search results, duplicates were removed, and two authors (SS and BB) jointly screened titles and abstracts according to predefined eligibility criteria. Disagreements or doubts about the eligibility of articles were resolved through discussion with a third author when needed.

Studies were included if they met the following criteria:

1) adults with a mean age ≥45 y and an age distribution not extending <40 y (first SD), in alignment with prior methodological standards [15];
2) randomized controlled trials (RCTs) using a clearly defined fasting intervention (TRE, ADF, 5:2 periodic fasting, or religious fasting), consistent with the Cell consensus terminology [2], with or without a PA component;
3) a control group (CONT) maintaining HD or receiving a clearly defined controlled diet (e.g., CER);
4) intervention duration ≥4 wk, considered the minimum timeframe for meaningful physiologic changes [26,27];
5) assessment of muscle mass using validated body-composition methods (e.g., dual-energy X-ray absorptiometry, DEXA; bioelectrical impedance analysis, BIA; MRI; computed tomography; ultrasound) [28,29].

Studies were excluded if participants had medical conditions associated with primary muscle loss, e.g., dementia, cancer, end-stage kidney disease, liver failure, severe respiratory disease, dysphagia, eating disorders, bariatric surgery, terminal illness, AIDS/HIV, rheumatoid arthritis, or heart failure.

### Selection of sources of evidence

Two authors independently screened full texts of all potentially eligible studies. Reasons for exclusion were recorded. A third author was involved in all cases where there was doubt about the eligibility of the article. Consensus was reached through discussion without the need for arbitration by additional authors.

### Data extraction

A standardized data-charting form was developed by two authors based on clinically relevant variables. The form was piloted on the first 5 eligible studies and refined by mutual agreement. One author performed data extraction using an electronic spreadsheet, and a second author verified all entries. Disagreements or inconsistencies were resolved by discussion or consultation with a third author. Corresponding authors were contacted when clarification or missing data were required.

Extracted data included:

1) study design characteristics (randomization method, number recruited, number completing, dropout rate and reasons, sample size justification, study setting);
2) baseline participant characteristics, i.e., age, sex distribution, ethnicity, country, mean weight, BMI), MS parameters, disease conditions;
3) intervention characteristics (fasting type and schedule, control diet, diet quality including recommended energy, protein, carbohydrate, lipid and fiber intake, dietary pattern, adherence measures, visit frequency, strategies to promote compliance, adverse effects, presence and type of PA intervention, PA intensity, sessions/week and duration, monitoring techniques; energy expenditure);
4) muscle mass outcomes, i.e., FFM or lean mass in kg, appendicular skeletal muscle index (ASMI), FFM index, appendicular lean mass (ALM), ALM/BMI;
5) body-composition outcomes, i.e., BW, FM, visceral fat mass (VFM), WC;
6) secondary outcomes i.e., QoL using validated instruments; functional performance such as handgrip strength or gait speed; fasting insulin levels; markers related to insulin sensitivity and insulin resistance such as HOMA-IR or Quantitative Insulin Sensitivity Check Index (QUICKI).

Consistent with recent methodological standards [30,31], FFM and lean mass were treated as equivalent constructs, as reported in the primary studies.

### Data synthesis and analysis

Data synthesis was conducted using R software version 4.1.2 (R Foundation for Statistical Computing, Vienna, Austria) and the *metaphor* package. For studies reporting mean changes with SEs or 95% confidence intervals (CIs), between-group mean differences were calculated as the difference in change scores between intervention and comparator groups. The SE of the mean difference was derived assuming independent groups using the square root of the summed squared SEs. Corresponding 95% CIs were computed as the mean difference ± 1.96 × SE, and statistical significance was defined as CIs not crossing zero.

Any quantitative syntheses are presented exclusively in the Supplementary Materials and should be interpreted as exploratory and hypothesis-generating. Consistent with the PRISMA-ScR framework, the main manuscript adopts a descriptive evidence-mapping approach and does not rely on pooled estimates for interpretive conclusions. Furthermore, the exploratory meta-analysis performed (Supplementary material 2) does not include a formal risk-of-bias assessment or key muscle-related confounders (e.g., protein intake and structured exercise). Also, the high heterogeneity undermines the interpretability of pooled estimates, even in exploratory analyses, suggesting that these results reflect direction of effect rather than magnitude.

## Results

### Overview of the eligible studies

Figure 3 shows the PRISMA flow chart of the literature search and study selection. Initially, 8362 articles were identified, and 47 additional articles were identified through snowballing and reference screening. After removing duplicates, 3876 articles underwent title/abstract screening. Full-text screening was then performed for 64 papers retrieved from databases/registries and 30 from other methods. Ultimately, 28 articles were included, providing results from 20 RCTs [[32], [33], [34], [35], [36], [37], [38], [39], [40], [41], [42], [43], [44], [45], [46], [47], [48], [49], [50], [51], [52], [53], [54], [55], [56], [57], [58], [59]].

**FIGURE 4:**
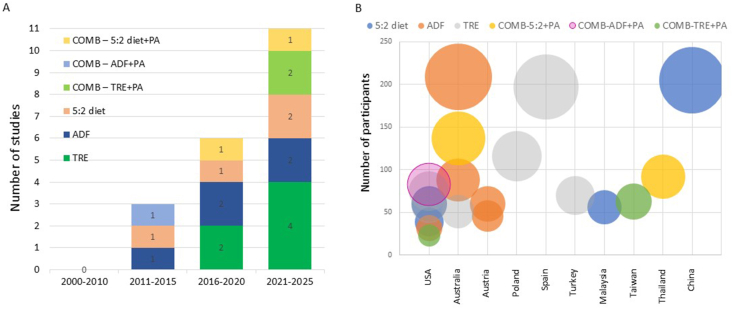
Number of studies per year range (A) and distribution of studies per countries (B). ADF, alternate-day fasting; COMB, combination of fasting and physical activity; PA, physical activity; TRE, time-restricted eating.

Figure 4A highlights the rise in popularity of fasting studies in middle-aged adults and older adults within the last decade. Indeed, none of the eligible studies was conducted in the period 2000–2010. 3 studies were published between 2011 and 2015, 6 between 2016 and 2020, and 11 between 2021 and 2025. A similar trend was also observed for TRE as a fasting intervention type.

Studies were conducted across diverse geographic settings, including Australia (4 studies; 477 participants), Austria (2 studies; 106 participants), the United States (7 studies; 320 participants), and single studies from China, Malaysia, Poland, Spain, Taiwan, Thailand, and Turkey. The highest number of participants were from Australia, United States, China, and Spain (Figure 4B).

### Participants characteristics

Table 1 presents the key characteristics of the included studies [[32], [33], [34], [35], [36], [37], [38], [39], [40], [41], [42], [43], [44], [45], [46], [47], [48], [49], [50], [51], [52], [53], [54], [55], [56], [57], [58], [59]]. In total, 1653 middle-aged and older adults completed the 20 RCTs. Participants aged from 40 to 70 y (mean ± SD: 53.3 ± 9.6).

**TABLE 1:** Characteristics of included studies

First author (y)	Study design	Country	Study setting	Baseline population description	Baseline number of participants (INT; CONT)	Baseline age (mean ± SD or range)	Baseline number of females (%)	Baseline ethnicity (%)	Baseline mean BMI (mean ± SD), kg/m^2^	Baseline metabolic syndrome (yes/no) [ref]	Baseline cardiometabolic diseases	Insulin levels	Insulin sensitivity/insulin resistance
Teng NI (2013) [32]	RCT (2 parallel arms, 1:1)	Malaysia	Community	Middle-aged adults normal weight or overweight	56 participants (28 in INT; 28 in CONT)	59.6 ± 5.4 y in INT; 59.1 ± 6.2 in CONT	0	—	26.8 ± 1.7 in INT; 26.7 ± 2.3 in CONT	Yes (TG∗∗∗, BP, FBG)	Hypertension	—	—
Panizza CE (2019) [33]	RCT (2 parallel arms, 1:1)	United States	Community	Middle-aged adults, with overweight or obesity	60 participants (30 in INT; 30 in DASH)	48.4 ± 4.7 y old in INT; 46.2 ± 5.4 in CONT	70	15% Chinese, 60% Japanese, 11,7% Korean, 13,3% Mixed Asian	30.5 ± 3.5 in INT; 30.8 ± 3.3 in DASH	Yes (WC, HDL, SBP, FBG)	Dyslipidemia, high blood pressure	ns difference between groups; s improved in INT and CONT	—
Stekovic S. (2019); Tripolt N.J. (2018) [34,35]	RCT (2 parallel arms, 1:1)	Austria	Primary care and community	Middle-aged adults with overweight	60 participants (30 in ADF, 30 in CONT)	48 y old (42.5–55) in ADF; 50.5 (44.5–56.75) in CONT	56.7	—	25.51 ± 1.80 in ADF; 25.37 ± 2.16 in CONT	No	No	—	ns differences in HOMA-IR, QUICKI and Matsuda between ADF and CONT
Varady KA (2013) [36]	RCT (2 parallel arms, 1:1)	United States	Research center	Middle-aged adults with overweight	32 participants (16 in INT, 16 in CONT)	47 ± 3 y old in INT; 48 ± 2 in CONT∗	68.8∗	40.6% African American; 43,8% Caucasian; 9.4% Hispanic; 6.2% others∗	26 ± 1 in INT; 26 ± 1 in CONT∗	No	No	—	—
Obermayer A (2023); Obermayer A (2022) [37,38]	RCT (2 parallel arms, 1:1)	Austria	Hospital and community	Older adults with a long duration of diabetes and obesity	46 participants (22 in INT; 24 in CONT)	63 ± 7 y old	47.8	—	34.3 ± 4.5	Yes (WC∗∗, SBP, FBG)	Type 2 diabetes, hypertension, dyslipidemia	—	—
Cienfuegos S. (2020) [39]	RCT (3 parallel arms, 1:1:1)	United States	Community	Middle-aged adults with obesity and with insulin resistance	58 participants (19 in 4-h TRE, 20 in 6-h TRE, 19 in CONT)	47 ± 2 in 4-h TRE; 47 ± 3 in 6-h TRE; 45 ± 2 in CONT	91.4	10.3% White; 63.8% Black; 8.6% Asian; 15.5% Hispanic; 1.7% other	37 ± 1 in 4-h TRE; 37 ± 1 in 6-h TRE; 36 ± 1 in CONT	No	Hypertension	s reduced in 4-h and 6-h TRE vs. CONT	s reduced HOMA-IR in 4-h and 6-h TRE vs. CONT
Domaszewski P. (2023) [40]	RCT (2 parallel arms, 1:1)	Poland	Community	Older adults with overweight	116 participants (61 in TRE, 55 in CONT)	TRE: 69.7 ± 3.11 for females; 68.1 ± 3.83 for males. CONT: 68.8 ± 3.45 for females; 68.8 ± 3.44 for males	50.9	—	TRE: 28.7 ± 3.99 in females; 27.8 ± 1.79 in males. CONT: 27.3 ± 3.79 in females; 27.9 ± 1.72 in males	No	No	—	—
Parr EB (2024) [41]	RCT (2 parallel arms, 1:1)	Australia	Research center	Middle-aged adults with type 2 diabetes and overweight or obesity	51 participants (26 in INT, 25 in CONT)	55.9 ± 8.1 y old	41	45% Caucasian, 24% European, 29% Asian, 2% Hispanic	33.0 ±4.7	Yes (WC∗∗, HDL, diabetes)	Type 2 diabetes	ns difference in the reduction between groups	ns difference in the HOMA-IR reduction between groups
Dote-Montero M (2025); Dote-Montero M (2024) [42,43]	RCT (4 parallel arms, 1:1:1:1)	Spain	Community	Middle-aged adults with overweight and obesity	197 participants (49 in eTRE; 52 in lTRE; 47 in ssTRE; 49 in CONT)	47.2 ± 6.2 y in eTRE; 48.0 ± 6.9 in lTRE; 45.2 ± 5.8 in ssTRE; 46.7 ± 6.0 in CONT	49.7	—	33.4 ± 3.7 in CONT; 33.8 ± 3.3 in eTRE; 32.4 ± 3.4 in lTRE; 32.4 ± 3.3 in ssTRE	No	No	ns difference between groups	ns difference between groups in HOMA-IR; s reduction in the eTRE
He CJ (2021) [44]	RCT (2 parallel arms, 1:1)	China	Research hospital and local community	Middle-aged adults with overweight or obesity, with hypertension	205 participants (102 in INT; 103 in CER)	50.5 ± 8.8 y old	57.6	—	28.7 ± 2.7	Yes (WC, HDL, TG, FBG)	Hypertension, diabetes	—	—
Kunduraci Y (2020) [45]	RCT (2 parallel arms, 1:1)	Turkey	Research clinic	Middle-aged adults with overweight	70 participants (35 in INT, 35 in CER)	47.44 ± 2.17 y in INT; 48.76 ± 2.13 in CER∗	52.3∗	—	36.58 ± 0.93 in INT; 32.82 ± 0.72 in CONT∗	Yes (according to IDF 2005 or NCEP-ATP III)	No	ns differences between groups; s reduction in CER	ns differences between groups; s reduction in HOMA-IR in INT and CONT at 12 weeks
Teong X.T. (2023); Teong X.T. (2021); Teong X.T. (2020) [[46], [47], [48]]	RCT (3 parallel arms, 2:2:1)	Australia	Community	Middle-aged adults and older adults with overweight or obesity, nondiabetic	209 participants (85 in TRE, 83 in CER, 41 in CONT)	57 ± 10 y old in TRE; 58 ± 10 in CER; 59 ± 11 in CONT	57.4§	—	34.7 ± 4.6 in TRE; 35.0 ± 4.6 in CER; 33.8 ± 4.9 in CONT	No	Impaired fasting blood glucose (patients with high risk of developing diabetes)	ns differences between groups; s reduction in postprandial insulin AUC in TRE vs. CER and CONT at 6 months	ns differences in Matsuda and insulinogenic index between groups
Hutchison A.T. (2019); Teong X.T. (2021) [49,50]	RCT (4 parallel arms, 1:1:1:1)	Australia	Community	Middle-aged adults with overweight or obesity	88 participants (25 in IF70, 25 in IF100, 26 in CER, 12 in CONT)	50 ± 1 y old	100	—	32.3 ± 0.5	No	No	— (reported just after a fed or fast day)	— (reported just after a fed or fast day)
Pavlou V (2023) [51]	RCT (3 parallel arms, 1:1:1)	United States	Community	Middle-aged adults, with obesity and high hba1c	75 participants (25 in TRE, 25 in CER, 25 in CONT)	55 ± 12 y old	71	40% Hispanic White, 53,3% Non-Hispanic Black, 5,3 Non-Hispanic White, 1,2% Asian	39 ± 7	Yes (WC, TG, BP, FBG)	Type 2 diabetes	—	—
Keawtep P (2024); Keawtep P (2023) [52,53]	RCT (4 parallel arms, 1:1:1:1)	Thailand	—	Middle-aged adults with obesity	92 participants (23 in DIET INT; 23 in EX INT; 23 in COMB; 23 in CONT)	52.87 ± 3.88 y old in DIET INT; 52.70 ± 3.60 in EX INT; 52.17 ± 3.35 in COMB; 53.61 ± 2.81 in CONT	100	—	28.28 ± 2.78 in DIET INT; 29.06 ± 2.90 in EX INT; 29.73 ± 4.57 in COMB; 29.18 ± 2.85 in CONT	No possible conclusion based on the available data	Hypertension, hypercholesterolemia, type 2 diabetes	s reduction in EX and COMB groups vs. CONT; ns difference among COMB, DIET and EX groups	s reduction in EX and COMB groups vs. CONT; ns difference among COMB, DIET and EX groups
Bhutani S (2013) [54]	RCT (4 parallel arms, 1:1:1:1)	United States	Research center	Middle-aged adults with obesity	83 participants (18 in COMB, 25 in ADF, 24 in PA, 16 in CONT)	45 ± 5 y old in COMB; 42 ± 2 in ADF; 42 ± 2 in PA; 49 ± 2 in CONT	96.4	49.4% African American, 25.3% Caucasian, 21.7% Hispanic, 3.6% other	35 ± 1 in COMB; 35 ± 1 in ADF; 35 ± 1 in PA; 35 ± 1 in CONT	No	No	ns difference in any intervention group	ns difference in HOMA-IR in any intervention group
Kotarsky, C.J. (2021) [55]	RCT (2 parallel arms, 1:1)	United States	Community	Middle-aged adults, mostly females, with overweight	23 participants (11 in INT, 10 in CONT)	45 ± 3 y old in INT; 44 ± 2 in CONT∗	85.7∗	—	29.8 ± 0.8 in INT; 29.4 ± 0.8 in CONT∗	No	No	ns differences between and within groups	—
Lin YJ (2022) [56]	RCT (2 parallel arms, 1:1)	Taiwan	Community	Middle-aged adults with overweight	63 females (30 in INT; 33 in CER)	50.1 ± 7.5 y in INT; 54.2 ± 7.9 in CER	100	—	25.9 ± 3.7 in INT; 25.7 ± 3.8 in CER	No	No	ns difference between groups; baseline fasting insulin was s higher in CONT vs. TRE	s increase in HOMA-IR in INT; baseline HOMA-IR was s higher in CONT vs. TRE
Carter S (2018) [57]	RCT (2 parallel arms, 1:1)	Australia	Community	Older adults with obesity	137 participants (70 in INT; 67 in CER)	61 ± 9.1 y old	56.2	—	36 ± 5.8	Yes (WC∗∗, BP, FBG)	Type 2 diabetes	—	—
Arciero et al., 2022; Arciero et al., 2023 [58,59]	RCT (2 parallel arms, 1:1)	United States	Community	Middle-aged adults with obesity	41 participant: IF-P (*n* = 21) and CER (*n* = 20)	49.7 ± 2.1 y in IF-P; 50.7 ± 2.4 in CER	66.7∗	—	32.4 ± 1.7 in IF-P, 33.0 ± 1.6 in CER	No	Hypercholesterolemia	Baseline fasting insulin was ns higher in IF-P vs. CER	—

Marks: ∗ if data are just for completers; ∗∗ assumption that waist circumference is high given that BMI ≥ 30 kg/m^2^; ∗∗∗ wide triglyceride range and statistically significant difference between-study arms; § at 6 months, 35/69 (51%) of the participants decided to reduce the number of iTRE days to twice per week (just 1 participant 1/wk) and CER were given new target calories that increased by 10%–15% above their current plan.

Abbreviations: ADF, alternate-day fasting; CER, caloric energy restriction; COMB, combined; CONT, control; DASH, Dietary Approaches to Stop Hypertension; DR, dietary restriction; EX, exercise; IF, intermittent fasting; INT, intervention; ns, not significant; PA, physical activity; QUICKI, Quantitative Insulin Sensitivity Check Index; RCT, randomized controlled trial; s, significant; TRE, time-restricted eating; WC, waist circumference; SBP, Systolic Blood Pressure; FBG, Fasting Blood Glucose; eTRE, early Time Restricted Eating; lTRE, late Time Restricted Eating; IDF, International Diabetes Federation; NCEP-ATP, National Cholesterol Education Program - Adult Treatment Panel; TG, triglycerides; BP, blood pressure; HbA1c, glycated haemoglobin; IF-P, Intermittent Fasting with protein pacing.

Although this review included studies in middle-aged and older adults, the evidence base was weighted toward middle-aged and early older adulthood. Several studies categorized participants as older adults despite mean ages in the 60s, and specifically few trials enrolled adults aged ≥70 y [37,40,57]. Therefore, findings should not be generalized to geriatric populations, frail older adults, or individuals at high risk of sarcopenia.

The overall participants were predominantly female (64%). Three studies included only females [49,52,56], and 1 included only males [32].

Participants had varying health conditions including overweight or obesity (all studies), type 1 or type 2 diabetes (5 studies), hypertension (5 studies), and dyslipidemia (2 studies). Using the international consensus of Alberti et al. [60], MS was identifiable in 8 studies (637 participants). Where MS elements were not fully reported, MS classification was based on available components, and studies with insufficient data were noted.

### Intervention compared with control details

Regarding fasting interventions, TRE was the most common (8 studies), followed by ADF (7 studies) and the 5:2 diet (5 studies) (Figure 4A). No eligible trials of Ramadan or other religious fasting met inclusion criteria, but one 5:2 diet study relied on principles of religious fasting [32]. The 8-h eating window between 12:00 and 20:00 was the most common form of TRE (3 studies, [40,51,55]). Participants were allowed to choose between predefined windows (2 studies, [45,56]), or were allocated to early, late, or self-selected fasting arms (1 study, [42]). Additionally, 4-and 6-h [39], as well as 9-h windows [41] were also used (1 study each). Most CONT groups followed exclusively HD (9 studies [[32], [33], [34], [35], [36], [37], [38], [39], [40], [41], [42],^54^,^55^]), or exclusively CER (5 studies [44,45,56,57,59]) as the comparator. In 2 studies, both HD and CER were involved [49,51]. Intervention types were classified according to the 2024 consensus terminology [2]. The detailed information on the study design and fasting regimens is provided in Table 1 and Table 2. Study implementation and adherence can be found in Supplementary Тable 1.

**TABLE 2:** INT vs. CONT description

First author (year)	INT	INT description		CONT	CONT description	Nutritional guidelines/protocol referenced	Duration	Follow-up period	Muscle mass assessment technique
Teng et al., 2013 [32]	5:2 diet (+CER)	IF combined with caloric restriction: 2 d of fasting for approx. 13 h/d (1 meal before sunrise and 1 meal after sunset) and 5 feeding days with 300–500 kcal reduction/d. Nutritional composition was not specified.		HD	HD; participants maintained HD and lifestyle	2010 Malaysian Dietary guideline	12 weeks	No	BIA
Panizza et al., 2019 [33]	5:2 diet	Two consecutive fasting days with 70% energy restriction: 34%, 33%, and 33% of energy from protein, carbohydrate, and fat intakes. Five feeding days: euenergetic MED diet; 25%, 45%, and 30% of energy from protein, carbohydrate, and fat.		DASH diet	Euenergetic DASH diet; with no caloric restriction	—	12 weeks	Yes, after 9 months	DEXA
Stekovic et al., 2019; Tripolt et al., 2018 [34,35]	ADF	Eating every second day ad libitum, but refraining from calorie intake on the fast days by completely excluding solid and liquid foods and caloric beverages (including diet sodas and calorie-free meals or beverages; tea and coffee allowed)		HD	HD participants maintained their HD and lifestyle	—	4 weeks	Yes, after 2 years	DEXA
Varady et al., 2013 [36]	ADMF	Modified ADF: low energy diet (25% of baseline energy needs, 24 h) on fasting days and ad libitum eating on each alternating feed day (24 h). Subjects were provided with fast day meals (400–600 kcal, 30% kcal from fat, 15% kcal from protein, 55% kcal from carbohydrate), consumed between 12:00 and 14:00. Energy-free beverages, tea, coffee, and sugar-free gum were allowed.		HD	HD participants were permitted to eat ad libitum every day, and were not provided with meals.	AHA guidelines	12 weeks	No	DEXA
Obermayer et al., 2023; Obermayer et al., 2022 [37,38]	ADMF	Modified IF: 3 d a week of fasting by consuming 25% of the recommended caloric intake (ingestion was only allowed at breakfast and/or lunch to maintain an 18-h period of fasting); Other 4 d a week ad libitum eating. There was no restriction on macronutrient composition or consumption of water, unsweetened coffee, and tea without milk.		HD	HD; maintain eating and lifestyle behavior	German Nutrition Society guidelines	12 weeks	No	BIA
Cienfuegos et al., 2020 [39]	TRE (4-h TRE)	Time-restricted eating without caloric restriction (15:00–19:00 h); energy-free beverages allowed		HD	HD; participants maintained their HD and lifestyle with ad libitum food intake and no time restriction	—	8 weeks	No	DEXA
	TRE (6-h TRE)	Time-restricted eating without caloric restriction (13:00–19:00 h); energy-free beverages allowed							
Domaszewski et al., 2023 [40]	TRE (8-h TRE)	Time-restricted eating without caloric restriction (00:00–20:00 h); beverages with very low or zero calorie content were allowed during fasting.		HD	HD and an educational program on healthy eating habits	—	6 weeks	No	BIA
Parr et al., 2024 [41]	TRE (9-h TRE)	Time-restricted eating without caloric restriction (10:00–19:00 h)		HD∗	individualized dietetic guidance—for type 2 diabetes	Australian dietary guideline	24 weeks	No	DEXA
Dote-Montero et al., 2025; Dote-Montero et al., 2024 [42,43]	TRE (8-h eTRE)	8-h early time-restricted eating without caloric restriction; starting no later than 10:00 h (10:00–18:00 h)		HD	HD; //All groups received an educational program for weight management and cardiovascular health promotion based on the MED dietary pattern and the WHO recommendations for PA	Mediterranean (MED) dietary patterns	12 weeks	No	DEXA
	TRE (8-h lTRE)	8-h late time-restricted eating without caloric restriction; starting not before 13:00 h (13:00–21:00 h)							
	TRE (8-h ssTRE)	8-h self-selected time-restricted eating window; without caloric restriction							
He et al., (2021) [44]	5:2 diet	IF: 2 d with very-low calories diet (500–600 kcal/d/5 d) ad libitum eating; minimum of 0.8 g supplemental of protein per kilogram of body weight per day; not specified the nutritional composition		CER70	Diet of 1000 kcal/d for females and 1200 kcal/d for males	Dietary Chinese guidelines, general principles of a MED diet	6 months	No	DEXA
Kunduraci et al., (2020) [45]	TRE (8-h TRE +CER)	8-h TRE, with energy-restricted diet (75% of energy requirements daily), not defined a specific time range; Not specified the nutritional composition. Sugar-free tea and black coffee allowed in fasting		CER75	75% energy requirements daily	Turkey National Dietary Guidelines	12 weeks	No	BIA
Teong et al., (2023); Teong et al., (2021); Teong et al., (2020) [[46], [47], [48]]	ADMF	Modified ADF: 30% of energy requirements taken between 08:00 h and 00:00 h, followed by 20-h fasting period on 3 nonconsecutive days per week, combined with ad libitum eating on other 4 d. Prescribed menu included 2 meal replacements at breakfast and lunch. Not specified the nutritional composition.		SC	Standard care –booklet guidelines on achieving and maintaining a healthy weight, such as healthier food choices and portion control.	- 2013 AHA/ACC/TOS guideline for the management of overweight and obesity in adults - 2013 Australian Dietary Guidelines	24 weeks	Yes, after 12 months	DEXA
				CER70	70% energy requirements daily; The macronutrient distribution of the menu plans was based on on a typical Australian dietary pattern (∼50% carbohydrate, 20%protein and 30% fat).				
Hutchison et al., (2019); Teong et al., (2021) [49,50]	ADMF +CER70	ADF with caloric restriction; fasting for 24 h after breakfast on 3 nonconsecutive days per week/eating with 70% of calculated baseline energy requirements on other days.	Diet drinks, chewing gum, black coffee, tea, very-low-energy broth were allowed in fasting. All diets were matched for macronutrient composition (35% fat, 15% protein, 50% carbohydrate).	HD	HD; continuous energy intake at 100% of baseline energy (no caloric restriction)	2013 AHA/ACC/TOS guideline for the management of overweight and obesity in adults	8 weeks	No	DEXA
	ADMF	ADF without caloric restriction; fasting for 24 h after breakfast on 3 nonconsecutive days per week/eating with no caloric restriction on other days per week.		CER70	70% energy requirements daily				
Pavlou et al., (2023) [51]	TRE (8-hTRE)	TRE without caloric restriction (00:00–20:00 h); no restrictions on types and quantities of foods consumed; energy-free drinks allowed		HD	HD; maintaining usual eating and lifestyle habits.	ADA nutrition guidelines	24 weeks	No	DEXA
				CER75	75% energy requirements daily				
Keawtep et al., 2024; Keawtep et al., 2023 [52,53]	5:2 diet	IF: 2 d of caloric restriction/5 d of ad libitum eating; self-selected dietary intake with 25%–75% of their estimated energy requirements for 2 d/wk (75% of energy requirements for weeks 1–4, 50% of energy requirements for weeks 5–8, and 25% of energy requirements for weeks 9–12) and ad libitum eating on the remaining 5 d		HD	To continue daily routine activities and usual lifestyle behavior without any prescribed PA or diet	—	12 weeks	No	BIA
	COMB = 5:2 diet + PA	IF (2 d of caloric restriction/5 d of ad libitum eating) + PA 60 min/d, 3 d/wk on ad libitum days of the dietary intervention		PA	Exercise group (physical-cognitive exercise 60 min/d, 3 d/wk)				
Bhutani et al., 2013 [54]	ADMF	Modified ADF: fasting days: low energy diet (25% of baseline energy needs) with only 1 meal consumed between 12:00 h and 14:00 h on the fast days/feeding days: ad libitum eating. Fast day meal: 450 kcal per fast day: 11–13 g of fats (22%–26% of energy); 25–29 g of proteins (22%–26% of energy); 60 g of carbohydrates (52% of energy), 10 g fiber.		HD	HD; maintained usual food and lifestyle habits	None	12 weeks	No	BIA
	COMB = ADMF + PA	Modified ADF: low energy diet (25% of baseline energy needs) between 12:00 h and 14:00 h on the fast days/ ad libitum eating on feeding days (24 h) + moderate training 3 d/wk		PA	Moderate training 3d/wk; ad libitum number of meals everyday				
Kotarsky et al., (2021) [55]	COMB = TRE (8-h TRE) + PA	Time-restricted eating without caloric restriction (12:00–20:00 h); black coffee and tea allowed + resistance training on nonconsecutive days		HD+PA	HD + PA (resistance training on nonconsecutive days)	—	8 weeks	No	DEXA
Lin et al., (2022) [56]	COMB = TRE (8-hTRE) + PA	TRE with caloric restriction (10:00–18:00 h or 00:00–20:00 h); tea with no sugar and black coffee allowed in fasting. 1400 kcal/d; Not specified the nutritional composition. +1 30-min exercise sessions/week		CER70+PA	1400 kcal/d; a traditional weight-loss method, eating 3 meals normally, without time restriction +1 30-min exercise session/week.	—	8 weeks	No	BIA
Carter et al., (2018) [57]	5:2 diet+PA	IF; 2 nonconsecutive days of caloric restriction with 500–600 kcal/d; and 5 d of ad libitum eating. Min. 50 g of protein/d. +PA: walking; all participants were advised to increase their step count by 2000 and maintain this increase over the trial.		CER70 +PA	Continuous energy restriction diet of 1200 to 1500 kcal/d (30% protein, 45% carbohydrate, and 25% fat); the same walking recommendation as INT	VLCDs guideline	12 months	Yes, after 24 months	DEXA
Arciero et al., (2022); Arciero et al., (2023) [58,59]	IF-P	IF for 1 d (IF 36 h total) or 2 consecutive days (IF 60 h total) a week plus protein pacing (P) in nonfasting days; P days for IF1-P consisted of 4 and 5 meals/d providing 1350 and 1700 kcal/d for females and males, respectively, and a macronutrient distribution targeting 35% protein, 35% carbohydrate, and 30% fat. IF2-P followed a similar P meal protocol providing 1500 and 1850 kcal/d for females and males, respectively, and similar macronutrient distribution and total weekly calorie intakes (8500 kcal/wk) as IF1-1 for weeks 1 through 4. Thereafter, IF1-P and IF2-P followed identical meal plans. On a weekly basis, caloric intake was the same for both intervention (IF-P) and control (CER70) groups.		CER70	Heart-healthy continuous energy restriction diet of 1200 kcal/d (for females) and 1500 kcal/d (for males); The macronutrient distribution: <35% fat; 50% to 60% carbohydrates; <200 mg/dL of dietary cholesterol; 20 to 30 g/d of fiber; and low sugar intake (<50 g/d)	National Cholesterol Education Program Therapeutic Lifestyle Changes (TLC) diet of the AHA	8 weeks	No	BODPod (for total FM and FFM), DEXA for AF, VF, and subcutaneous AF mass to limit exposure to radiation

Abbreviations: ADF, alternate-day fasting; ADMF, alternate-day modified fasting; AF, abdominal fat; AHA, American Heart Association; BIA, bioelectrical impedance analysis; CER, caloric energy restriction; CONT, control; DASH, Dietary Approaches to Stop Hypertension; DEXA, dual-energy X-ray absorptiometry; FFM, fat-free mass; FM, fat mass; HD, habitual diet; IF, intermittent fasting; INT, intervention; MED, Mediterranean diet; PA, physical activity; TLC, therapeutic lifestyle changes; TRE, time-restricted eating; VF, visceral fat, VLCD, very-low-calorie diets; ACC, American College of Cardiology; TOS, The Obesity Society; IF-P, Intermittent Fasting with protein pacing; BODPod, an air displacement plethysmograph system that uses densitometry principles to determine body composition.

The details on interventions and controls are described in Table 2. Across the 20 eligible studies, a total of 51 study arms were identified, i.e., 26 fasting-based intervention arms and 25 control arms. Although 6 studies are categorized as using a 5:2 diet [32,33,44,52,57,59], substantial heterogeneity was observed in their protocol design. Indeed, fasting days were consecutive [33] or nonconsecutive [32,44,52,57], whereas nonfasting days more commonly allowed ad libitum eating [44,52,57] or caloric restriction [32]. In Panizza et al. [33], participants followed Mediterranean diet principles in the nonfasting period [33]. Instead, ADF protocols were comparatively more uniform, with most studies employing an ADMF, allowing limited calorie intake (*ca.* 25% of baseline energy needs) at breakfast followed by a fasting period [36,37,46,49,54,59]. On feeding days, ad libitum eating was usually allowed. One study arm [49] restricted energy intake to 70% during nonfasting period. Additionally, the study of Arciero et al. [59] allowed IF for only 1 d/wk (36h in total) with caloric restriction combined with protein pacing at nonfasting days. TRE protocols also varied considerably, primarily with respect to the duration of the eating window, as described above. Their main limitation was that nutritional compositions of the diet were frequently not specified.

Control arms most commonly consisted of HD, i.e., participants were asked to maintain their usual eating patterns and lifestyle behavior, with no restriction in the eating window. However, the lack of detailed characterization or assessment of these habits complicates proper categorization and may introduce bias in the interpretation of results. HD was used as a control in 8 studies [32,[34], [35], [36], [37], [38], [39], [40], [41], [42]]. An isocaloric Dietary Approaches to Stop Hypertension diet served as control in 1 study [33], whereas CER was used in 3 studies [44,45,59]. Three studies included both HD and CER as control arms [49,51]. Twelve studies used established nutritional guidelines, mainly the national ones [32,36,37,41,[43], [44], [45], [46], [47],^51^,^57^,^59^], whereas in 8 studies this information was not addressed [33,34,39,40,52,[54], [55], [56]]. In 5 studies, participants were provided dietary protocols, i.e., specified the caloric and macronutrient compositions [33,36,54,57,59] and in 3 studies participants received educational program/booklets on healthy eating habits [40,42,46]. Detailed information is provided in Table 2.

Studies incorporating PA, referred to as combination of fasting and PA interventions (COMB), also exhibited considerable heterogeneity in design. Two of the 5 COMB studies employed 4-arm designs, separately evaluating fasting alone, fasting combined with PA, HD, and HD with PA [[52], [53], [54]]. The remaining 3 studies used 2-arm designs, with PA included in both intervention and control arms [[55], [56], [57]]. PA prescriptions varied widely, ranging from moderate-intensity resistance training performed 3 times per week [[52], [53], [54], [55]] to very-low–intensity activities, such as increases in daily step count in 1 study [57].

### Mapping of FFM outcomes

Table 3 summarizes changes in anthropometric and body-composition parameters across eligible studies, presenting differences in change from baseline to post-intervention between fasting and control groups.

**TABLE 3:** Body-composition parameters results

First author (year)	INT	CONT	duration	F (%)	MS	Assessment	FFM (kg)	FM (kg)	VFM (kg)	BW (kg)	WC (cm)
Teng et al., (2013) [32]	5:2 diet (+CER), *n=* 28	HD, *n =* 28	12 W	0	No	BIA	**1.1 (0.02, 2.18)**∗	**–1.2 (–1.54, –0.86)**∗	—	**–2.5 (–2.78, –2.22)**∗	—
Panizza et al., (2019) [33]	5:2 diet, *n =* 30	DASH, *n =* 30	12 W	70	Yes	DEXA	–1.0 (–5.99, 3.99) -total lean mass	–1.8 (–4.86, 1.26)	–11.8 (–29.67, 6.07) -VAT area (cm^2^)	–2.7 (–8.80, 3.40)	–2.5 (–6.93, 1.93)
Stekovic et al., (2019);Tripolt et al., (2018) [34,35]	ADF, *n =* 29	HD, *n =* 28	4 W	56.7	No	DEXA	**–0.81 (–1.11, –0.51)**∗-lean mass	**–3.81 (–4.18, –3.44)**∗	—	**–3.3 (–3.98,–2.63)**∗	—
Varady et al., (2013) [36]	ADMF, *n =* 15	HD, *n =* 15	12 W	68.8	No	DEXA	#not stat. diff.	**–3.6 (–4.70, –2.50)**∗	—	**–5.2 (–6.28, –4.12)**∗	—
Obermayer et al., (2023) and (2022) [37,38]	**ADMF**, *n =* 20	**HD**, *n =* 24	12 W	47.8	Yes	BIA	#not stat. diff.	**−3.6 (−5.14, −2.06)**∗	—	**−5.04 (−7.30, −2.78)**∗	—
Cienfuegos et al., (2020) [39]	**TRE** (4-h TRE), *n =* 16	**HD**, *n =* 14	8 W	91.4	No	DEXA	**–0.5 (–0.87, –0.13)**∗ -lean mass	**–2.2 (–3.00, –1.40)**∗	**–0.16 (–0.30, –0.02)**∗	**–3.3 (–4.12, –2.48)**∗	—
	**TRE** (6-h TRE), *n =* 19						**–1.2 (–1.76, –0.64)**∗ - lean mass	**–0.8 (–1.42, –0.18)**∗	–0.12 (–0.25, 0.01)	**–3.3 (–4.10, –2.50)**∗	—
Domaszewski et al., (2023) [40]	**TRE** (8-h TRE) *n* (F) = 29	**HD**, n (F) = 28	6 W	50.9	No	BIA	F: 0.2 (−1.78, 2.18)	—	F:0.02 (−0.41, 0.45)	F:−0.8 (−7.75, 6.15)	—
	**TRE** (8-h TRE) *n* (M) = 25	**HD**, n(M) = 26					M:−0.2 (−3.98, 3.58)	—	M:−0.62 (−1.38, 0.14)	M:−2.5 (−9.40, 4.40)	—
Parr et al., (2024) [41]	**TRE** (9-hTRE), *n =* 22	**HD**, *n =* 21	24 W	41	Yes, T2D	DEXA	0.2 (–0.36, 0.76) -lean mass	–0.2 (–0.99, 0.59)	–0.05 (–0.29, 0.19)	–0.5 (–1.48, 0.48)	—
Dote-Montero et al., (2025); Dote-Montero et al., (2024) [42,43]	**TRE** (8-h eTRE), *n =* 47	**HD**, *n =* 46	12 W	49.7	No	DEXA/ MRI	**FFM (kg):** −1.1(−2.6, 0.3) **ALM (kg)**: −0.9 (−1.9, 0.2)	−1.4(−3.0, 0.2)	–4 (–12, 4) -in % –75(–198, 48) cm^3^	**−2.9 (−4.7, −1.1)**∗	—
	**TRE** (8-h lTRE), *n =* 48						**FFM (kg):** −0.6(−2.1, 0.9) **ALM (kg)** −0.7 (–1.7, 0.4	−1.6(−3.2, 0.0)	–6 (–13, 2) -in % –74(–195, 47) cm^3^	**−2.4 (−4.2, −0.6)**∗	—
	**TRE** (8-h ssTRE), *n =* 42						**FFM (kg):** −1.2(−2.8, 0.3) **ALM (kg)** −0.7 (−1.8, 0.4)	−1.5(−3.2, 0.2)	–3 (–11, 5) -in % –73 (–198, 51) cm^3^	**−3.1 (−4.9, −1.2)**∗	—
He et al., (2021) [44]	**5:2 diet**, *n =* 88	**CER70**, *n =* 85	6 M	57.6	Yes	DEXA	**0.60 (0.03, 1.17)**∗	**−0.70 (−0.98, −0.42)**∗	—	0.10 (−0.40, 0.60)	—
Kunduraci et al., (2020) [45]	**TRE** (8-hTRE), *n =* 32	**CER75**, *n =* 33	12 W	52.3	Yes	BIA	**–1.04 (–1.97, –0.11)**∗	**–1.43 (–2.07, –0.79)**∗	—	**–2.46 (–3.49, –1.43)**∗	—
Teong et al., (2023, 2021, 2020) [[46], [47], [48]]	**ADMF**, *n =* 69	**SC**, *n =* 32	24 W	57.4	No	DEXA	–1.29 (–2.79, 0.21)	**–3.71 (–5.54, –1.88)**∗	**–0.25 (–0.43,–0.07)**∗	**−4.95 (−6.29, −3.61)**∗	**–4.89 (–4.95,–4.83)**∗
		**CER70**, *n =* 62					−0.52 (−1.46, 0.42)	−0.43 (−1.53, 0.67)	−0.03 (−0.20, 0.14)	−0.4 (−1.46, 0.66)	−0.36 (−2.24, 1.52)
Hutchison et al., (2019), Teong et al., (2021) [49,50]	**ADMF** (+CER70), *n =* 22	**HD**, *n =* 11	8 W	100	No	DEXA	**–1 (–1.39, –0.61)**∗	**–3.7 (–4.65, –2.75)**∗	—	**–5.8 (–6.77, –4.83)**∗	**–6.2 (–8.02, –4.38)**∗
		**CER70**, *n =* 24					**−0.80 (−0.98, −0.62)**∗	**−1.10 (−1.33, −0.87)**∗	—	**−1.50 (−1.76, −1.24)**∗	−**2.40 (−3.03, −1.77)**∗
	**ADMF**, *n =* 22	**HD**, *n =* 11					–0.1 (–0.46, 0.26)	**–2.1 (–3.07, –1.13)**∗	—	**–3.1 (–4.04, –2.16)**∗	**–2.9 (–4.53, –1.27)**∗
		**CER70**,*n =* 24					0.10 (−0.08, 0.28)	**0.50 (0.27, 0.73)**∗	—	**1.20 (0.94, 1.46)**∗	**0.90 (0.35, 1.45)**∗
Pavlou et al., (2023) [51]	**TRE** (8-hTRE), *n =* 23	**HD**, *n =* 24	6 M	71	Yes	DEXA	–0.38 (–0.91, 0.15) -lean mass	**–2.49 (–4.41, –0.57)**∗	–0.09 (–0.31, 0.13)	**–3.45 (–6.17, –0.73)**∗	**–3.44 (–5.71, –1.17)**∗
		**CER75**,*n =* 22					0.13 (–0.34, 0.60) -lean mass	–1.65 (–3.32, 0.02)	–0.11 (–0.29, 0.07)	–1.56 (–3.63, 0.51)	**–3.5 (–5.81, –1.19)**∗
Keawtep et al., (2024, 2023) [52,53]	**5:2 diet**, *n =* 21	**HD**, *n =* 20	12 W	100	—	BIA	−0.29 (−4.05, 3.47)	−2.25 (−8.27, 3.77)	—	−3.57 (−12.07, 4.93)	—
		**PA**, *n =* 19					−0.01 (−3.88, 3.86)	0.29 (−5.71, 6.29)	—	−0.68 (−9.09, 7.73)	—
	**COMB = 5:2 diet + PA**, *n =* 20	**HD**, *n =* 20					−0.12 (−4.71, 4.47)	−3.36 (−11.7, 4.98)	—	−3.74 (−13.68, 6.20)	—
		**PA**, *n =* 19					0.16 (−4.27, 4.59)	−0.82 (−8.51, 6.87)	—	−0.85 (−10.7, 9.0)	—
Bhutani et al., (2013) [54]	**ADMF**, *n =* 16	**HD**, *n =* 16	12 W	96.4	No	BIA	0 (−4.5, 4.5)	−2 (−10.3, 6.3)	—	−3 (−14.4, 8.4)	−4 (−11.6, 3.6)
		**PA**, *n =* 16					0 (−4.5, 4.5)	−1 (−6.7, 4.7)	—	−2 (−9.1, 5.1)	−2 (−7.9, 3.9)
	**COMB =** ADMF+PA, *n =* 16	**HD**, *n =* 16					1 (−3.7, 5.7)	−5 (−21.0, 11.0)	—	6 (−6.6, 18.6)	−7 (−18.5, 4.5)
		**PA**, *n =* 16					1 (−3.0, 5.0)	−4 (−20.0, 12.0)	—	−5 (−17.0, 7.0)	−5 (−16.0, 6.0)
Kotarsky et al., (2021) [55]	**COMB** = TRE+PA, *n =* 11	**HD+PA**, *n =* 10	8 W	85.7	No	DEXA	**1 (0.72, 1.28)**∗ -lean mass	**–2 (–2.28, –1.72)**∗	**–0.07 (–0.11, –0.03)**∗	**–3.00 (–3.01, –2.99)**∗	**–2.2 (–2.48, –1.92)**∗
Lin et al., (2022) [56]	**COMB** = 8-hTRE+PA, *n =* 30	**CER70+PA**, *n =* 33	8 W	100	No	BIA	**–0.5 (–0.67, –0.33)**∗	**–0.5 (–0.61, –0.39)**∗**-in %**	0 (–0.10, 0.10)	**–1.1 (–1.47, –0.73)**∗	–0.3 (–1.06, 0.46)
Carter et al., (2018) [57]	**COMB = 5:2 diet**+PA, *n =* 51	**CER70**, *n =* 46	12 M	56.2	Yes	DEXA	–0.50 (–0.61, –0.61)	**−1.30 (−3.11, −0.49)**∗	0.00 (−0.25, 0.25)	−**1.80 (−4.02, −0.42)**∗	—
Arciero et al., (2022, 2023) [58,59]	**IF-P**, *n =* 20	**CER70**, *n =* 19	8 W	66.7	No	BODPod, DEXA	−0.5 (−8.3, 7.3)	−2.5 (−11.1, 6.1)	−0.3 (−1.1, 0.5)	−3.2 (−16.1, 9.7)	−3.3 (−13.2, 6.6)

Results are presented as the difference in change from baseline to postintervention between fasting and corresponding control group, expressed as MD, 95% CI. Details on INT and CONT are given in Table 2.

Abbreviations: ADF, alternate-day fasting; ADMF, alternate-day modified fasting; BIA, bioelectrical impedance analysis, BW, body weight; CER, caloric energy restriction; CI, confidence intervals; COMB, combination of fasting and physical activity; CONT, control; DASH, Dietary Approaches to Stop Hypertension; DEXA, dual-energy X-ray absorptiometry; F, female; FFM, fat-free mass; FM, fat mass; HD, habitual diet; INT, intervention; M, male; M, months; MD, mean difference; MS, metabolic syndrome; n, number of participants; PA, physical activity; TRE, time-restricted eating; T2D, type 2 diabetes; VFM, visceral fat mass, W, weeks; WC, waist circumference; BODPod, an air displacement plethysmograph system that uses densitometry principles to determine body composition; ALM, Appendicular Lean Mass.

∗ An asterisk denotes statistically significant differences. #The exact data could not be retrieved. In Varady et al. [36]—data were presented in form of graph, with a statement: “No difference between groups for fat-free mass at week 12”; in Obermayer et al. [37] was stated—“There was no statistically significant difference in the change in lean mass or bone mass between the 2 groups according to the DXA measurements.”

Across the included studies, FFM or lean mass outcomes were commonly reported alongside reductions in BW and FM during IF interventions. FFM was assessed in all 20 eligible studies. In line with established methodological standards [19,20,[27], [28], [29], [30]], FFM was considered equivalent to lean mass. FFM mass was assessed using DEXA in 12 studies, BIA in 7 studies, and BODPod (air displacement plethysmography) in 1 study. Given known methodological differences between DEXA and BIA, as well as historical variability in terminology related to FFM, the original terminology reported by study authors was retained in Table 3. The sensitivity of BIA to hydration status is also acknowledged as a potential source of measurement variability. Notably, only 1 study [42] reported muscle-specific indices such as ALM and provided additional data on ASMI and ALM/BMI by our request. Thus, synthesis of the results focused on total FFM (kg), rather than direct indicators of skeletal muscle preservation. Therefore, the evidence primarily reflects changes in whole-body composition and does not permit firm conclusions regarding muscle quality, sarcopenia-related processes, or long-term muscle preservation during aging.

Reported FFM responses varied considerably across fasting and control protocols. Positive between-group differences, indicating relatively greater FFM preservation in fasting interventions, were described in 10 of the 35 fasting compared with control comparisons. These findings were reported across several fasting approaches, including 5:2 diets, TRE combined with PA, and ADF interventions. Some studies described numerically larger FFM preservation during fasting interventions relative to HD or CER, although the magnitude of these differences was generally modest (∼1 kg; 32,44,54,55).

In contrast, most comparisons (23/35) described greater reductions in FFM during fasting interventions relative to controls. These findings were reported across multiple fasting protocols, including TRE, ADMF, and ADF interventions, and relative to both HD and CER groups. The reported between-group differences were typically small, often <1 kg. Several studies described larger numerical reductions in FFM, particularly in some ADMF and TRE interventions [39,45,46], although findings varied substantially across study designs, intervention duration, participant characteristics, and control conditions. In the remaining 2 comparisons, the exact values were not able to be retrieved [36,37], as explained in Table 3.

Nonetheless, interpretation is limited by substantial heterogeneity in fasting regimens, intervention duration, dietary composition, PA, and body-composition assessment methods. No uniformity among FFM outcomes was observed neither related to fasting regimens, nor to the comparator group (HD or CER group).

### Mapping of FM, BW, and anthropometric outcomes

Across studies, fasting interventions were most consistently associated with reductions in BW, FM, and WC. These patterns were observed across multiple fasting regimens, particularly ADF, ADMF, and several TRE protocols.

FM changes were assessed in 19 studies of the 20 eligible studies. Mapped studies commonly reported larger reductions in FM in fasting interventions than in both HD or CER controls. FM changes were greater in fasting than in control arm in 31 of the 33 comparisons, although the extent of change varied considerably between studies. The largest reductions in FM were reported in studies examining ADF or ADMF compared with HD controls [34,36,37,46], with reported differences generally ranging from approximately −3.6 to −3.8 kg. TRE-based interventions also frequently described reductions in FM relative to control conditions (∼–2.2 kg), including studies using 4- or 8-h eating windows [39,51], or combinations of TRE with PA [55]. Only 2 studies reported positive between-group differences in FM [49,52], whereas 1 study did not report data on FM [40].

VFM outcomes were inconsistently reported and were available in 11 of the 20 eligible studies. Mapped studies commonly reported larger reductions in fasting than in the comparator group [33,39,[40], [41], [42],^46^,^51^,^55^,^59^], although in 2 cases observed differences were small [39,46]. In 3 cases, no changes were observed [40,56,57]. Furthermore, it should be noted the inconsistency in reporting visceral fat results, being mainly presented as VFM (kg), but also as visceral adipose tissue (VAT) area (cm^2^) [33], or visceral fat volume (cm^3^) [42], which limits comparison among studies.

BW was assessed in all the studies. As expected, greater weight loss was observed in studies comparing fasting to HD rather than CER, and in studies incorporating PA. The overall directions of change in BW were similar to those reported for FM. The greatest reduction in BW (>3 kg greater loss in the fasting group than in controls) was recorded in the same studies as for the FM differences, mainly examining ADMF and TRE [36,37,39,46]. On the contrary, positive differences were reported for 3 studies, examining ADMF and 5:2 diet [44,49,54] and ranged from only +0.1 to +6 kg.

WC was assessed in only 8 studies, with changes ranging from –6.2 to +0.9 cm. Negative differences, i.e., higher WC losses in fasting intervention, were observed in 15 of 16 fasting compared with control comparisons, whereas positive difference was reported just once [49]. The most pronounced differences were reported in studies examining ADMF [46,49] and TRE [39], and most commonly when compared with HD.

Studies combining fasting with PA [52,54] also demonstrated larger reductions in FM, BW, and WC; however, these results were in the range with studies without PA.

Additionally, an exploratory meta-analysis (Supplementary Material 2) was performed for all anthropometric and body-composition outcomes. It included subgroup analyses based on control type (HD compared with CER, presented in Supplementary Figures 1–8) and presence of MS (presented in Supplementary Figures 9–13). However, these results should be interpreted cautiously due to the high heterogeneity, the absence of a formal risk-of-bias assessment, and the limited consideration of key muscle-related confounders (e.g., protein intake and structured exercise). These analyses were not intended to generate definitive pooled effects but to provide preliminary quantitative insights (hypothesis-generating) to guide future systematic reviews.

#### Results on diet quality (caloric and macronutrient intake, and timing)

To better distinguish whether observed body composition changes were attributable to metabolic effects or differences in energy balance and diet quality, data on dietary intake, including total energy intake and macronutrient composition, were extracted where available (Table 4). However, reporting of dietary intake was inconsistent across studies, limiting direct comparisons. Dietary intake data were available in 13 of the 20 included studies [[32], [33], [34], [35],^39^,^42^,[45], [46], [47], [48], [49], [50], [51],^55^,^56^,^59^], corresponding to 21 fasting compared with control comparisons. Four studies [[34], [35], [36],^42^] reported only total caloric intake without detailed macronutrient composition.

**TABLE 4:** Dietary intake results

First author (year)	INT	CONT	Energy (kcal/d)	Carbohydrate (g/d)	Fat (g/d)	Protein (g/d)	Fiber (g/d)
Teng et al. 2013 [32]	**5:2 diet** (+CER), *n =* 28	**HD**, *n =* 28	−366 (−589, −143)∗	−27.9 (−58.5, 2.7)	–28.9 (−42.1, −15.7)∗	−5.9 (−16.7, 4.9)	—
Panizza et al. 2019 [33]	**5:2 diet,***n =* 30	**DASH**, *n =* 30	−139 (−205, −73)∗	−48 (−56.1, −39.9)∗	2 (−1.45, 5.45)	5.6 (2.5, 8.7)∗	–1.4 (–2.13, –0.67)∗
Stekovic et al. 2019; Tripolt et al. 2018 [34,35]	**ADF**, *n =* 29	**HD**, *n =* 28	−3432 kcal/wk (−29.2%)†	—	—	—	—
Varady et al. 2013 [36]	**ADMF**, *n =* 15	**HD**, *n =* 15	64 (−329, 457)	—	—	—	—
Obermayer et al. 2023; 2022 [37,38]	**ADMF**, *n =* 20	**HD**, *n =* 24	—	—	—	—	—
Cienfuegos et al. 2020 [39]	**TRE** (4-h TRE), *n =* 16	**HD**, *n =* 14	–423 (–647.4, –198.6)∗	2 (−4.3, 8.3) (%)	−2 (−8.3, 4.3) (%)	0 (−2.8, 2.8) (%)	0 (−4.7, 4.7) (%)
	**TRE** (6-h TRE), *n =* 19		–461 (–757.4, –164.6)∗	−4 (−10.3, 2.3) (%)	0 (−6.3, 6.3) (%)	4 (1.2, 6.8)∗ (%)	3 (−7.4, 13.4) (%)
Domaszewski et al. 2023 [40]	**TRE** (8-h TRE) *n* (F) = 29	**HD**, n (F) = 28	Female: —	—	—	—	—
	**TRE** (8-h TRE), *n* (M) = 25	**HD**, n (M) = 26	Male: —	—	—	—	—
Parr et al. 2024 [41]	**TRE** (9-hTRE), *n =* 22	**HD**, *n =* 21	not possible to extract	—	—	—	—
Dote-Montero et al. 2025; [42,43]	**TRE** (8-h eTRE), *n =* 47	**HD**, *n =* 46	−307 (−570, −44)∗	—	—	—	—
	**TRE** (8-h lTRE), *n =* 48		−164 (−426, 98)	—	—	—	—
	**TRE** (8-h ssTRE) *n =* 42		−248 (−518, 21)	—	—	—	—
He et al. 2021 [44]	**5:2 diet**, *n =* 88	**CER70**, *n =* 85	—	—	—	—	—
Kunduraci et al. 2020 [45]	**TRE** (8-hTRE), *n =* 32	**CER75**, *n =* 33	−45.6(−287.3, 196.1)	7.23 (−25.4, 39.8)	−3.28 (−20.6, 14.0)	−10.05 (−24.99, 4.89)	−2.97 (−7.95, 2.01)
Teong et al. 2023, 2021, 2020 [[46], [47], [48]]	**ADMF**, *n =* 69	**SC**, *n =* 32	−280.9 (−413.6, −148.2)∗	−32.8 (−48.4, −17.2)∗	**−**12.5 (−19.1, −5.9)∗	−6.6 (−15.0, 1.8)	−4.2 (−7.5, −0.9)∗
		**CER70**, *n =* 62	−95.8(−190.8, −0.8)∗	−10.7 (−22.2, 0.8)	−2.8 (−7.5, 1.9)	−11.8 (−17.1, −6.5)∗	−4.7 (−6.6, −2.8)∗
Hutchison et al. 2019, Teong et al. 2021 [49,50]	**ADMF** (+CER70), *n =* 22	**HD**, *n =* 11	−9 (−146, 128)	−6 (−14.3, 2.3)	0 (−3.1, 3.1)	6 (1.9, 10.1)∗	—
	**ADMF**, *n =* 22		−34 (−116, 48)	−3 (−12.1, 6.1)	−1 (−4.5, 2.5)	−3 (−6.9, 0.9)	—
	**ADMF** (+CER70)	**CER70**, *n =* 24	−32 (−116, 52)	1 (−4.8, 6.8)	−1 (−2.6, 0.6)	−11 (−14.3, −7.7)∗	—
	**ADMF**		−25 (−111, 61)	3 (−2.8, 8.8)	−1 (−3.1, 1.1)	−9 (−12.3, −5.7)∗	—
Pavlou et al. 2023 [51]	**TRE** (8-hTRE), *n =* 23	**HD**, *n =* 24	−297 (−633, 39)	0 (−7.4, 7.4) (%)	−1 (−7.9, 5.9) (%)	1 (−3.8, 5.8) (%)	1 (−5.5, 7.5) (%)
		**CER75**, *n =* 22	−116 (−481, 249)	−2 (−8.5, 4.5)	1 (−6.4, 8.4)	1 (−4.4, 6.4)	0 (−7.8, 7.8)
Keawtep et al. 2024, 2023 [52,53]	**5:2 diet**, *n =* 21	**HD**, *n =* 20	—	—	—	—	—
			—	—	—	—	—
	**COMB = 5:2 diet + PA**, *n =* 20	**PA**, *n =* 19	—	—	—	—	—
			—	—	—	—	—
Bhutani et al. 2013 [54]	**ADMF**, *n =* 16	**HD**, *n =* 16	—	—	—	—	—
			—	—	—	—	—
	**COMB =** ADF + PA, *n =* 16	**PA**, *n =* 16	—	—	—	—	—
			—	—	—	—	—
Kotarsky et al. 2021 [55]	**COMB**=TRE + PA, *n =* 11	**HD + PA**, *n =* 10	−53 (−186, 80)	−34 (−49.8, −34 (−4	5 (2.23, 7.77)	−4 (−9.5, 1.5)	—
Lin et al. 2022 [56]	**COMB** = 8-hTRE + PA, *n =* 30	**CER70 + PA**, *n =* 33	−32.7 (−169.2, 103.8)	−12.3 (−37.5, 12.9)	1.0 (−5.6, 7.6)	−1.4 (−7.9, 5.1)	—
Carter et al. 2018 [57]	**COMB** = 5:2 diet + PA, *n =* 51	**CER70**, *n =* 46	—	—	—	—	—
Arciero et al. 2022, 2023 [58,59]	**IF-P**, *n =* 20	**CER70**, *n =* 19	94 (−165, 353)	−126 (−170, −82)∗	−50 (−69, −31)∗	62 (40, 84)∗	6 (−1.8, 13.8)

Results are presented as the difference in change from baseline to postintervention between fasting and corresponding control group, expressed as MD, 95% CI. Details on INT and CONT are given in Table 2.

Abbreviations: ADF, alternate-day fasting; ADMF, alternate-day modified fasting; CER, caloric energy restriction; CI, confidence intervals; COMB, combination of fasting and physical activity; CONT, control; DASH, Dietary Approaches to Stop Hypertension; HD, habitual diet; INT, intervention; MD, mean difference; n, number of participants; PA, physical activity; TRE, time-restricted eating; IF-P, Intermittent Fasting with protein pacing.

∗ An asterisk denotes statistically significant differences. †CI was not possible to calculate, as the results were presented as means ± SD or median (IQR) and no raw data were possible to retrieve. In Cienfuegos et al. [39] and Pavlou et al. [51], results are expressed as % of total energy intake.

Most studies reporting dietary intake described lower total energy intake during fasting interventions relative to HD and/or CER. Reductions in energy intake were observed across several fasting protocols, including 5:2 diets, TRE, and ADMF interventions. In contrast, 2 studies [36,59] described slightly higher caloric intake during fasting interventions relative to controls, coinciding with differing patterns in FM and BW outcomes.

Macronutrient intake reporting was heterogeneous across studies, both in methodology and units of presentation. Protein intake was the most frequently reported macronutrient outcome. Across 16 comparisons, several studies described stable or higher protein intake during fasting interventions, whereas others reported reductions relative to comparator groups. It is worth mentioning that the study by Arciero et al. [59] was focused on protein pacing, i.e., included increased protein intake on nonfasting days; thus, such a protein increase was intentional. Interpretation across studies was limited by inconsistent reporting formats, with some studies presenting protein intake as grams per day and others as a percentage of total energy intake.

Reporting of meal timing and actual eating windows was limited. Only 4 studies [41,42,55,56] provided data on actual eating windows followed by participants, and only 3 of these additionally reported eating windows for control groups [41,42,55]. Data on achieved eating windows and intervention adherence are summarized in Supplementary Table 1.

Overall, dietary intake reporting across studies was variable, and differences in caloric intake, protein intake, and eating-window adherence may have contributed to the heterogeneity observed in anthropometric and body-composition outcomes.

#### Results on insulin, insulin sensitivity and insulin resistance

Ten of the 20 studies reported insulin levels, whereas 9 assessed insulin sensitivity or resistance. Of note, among the 20 eligible studies, 6 enrolled participants with diabetes [37,41,44,51,52,57] and 1 included individuals at high risk of developing diabetes [46]. Notably, among the studies enrolling participants with diabetes, only 1 study (Parr et al.[41]) assessed both insulin levels and insulin sensitivity.

Regarding insulin levels, most studies reported comparable changes between fasting and control interventions [33,41,42,45,46,54,55,56]. In contrast, 2 studies [39,52] described larger reductions in insulin-related outcomes during fasting interventions, although in 1 study these findings were limited to the exercise (EX) and combined (COMB) intervention arms relative to controls [52].

Similarly, outcomes related to insulin sensitivity or insulin resistance, including HOMA-IR, QUICKI, the Matsuda index, and the insulinogenic index, were described in several studies [34,41,42,45,46,54,56]. Most reported comparable changes between fasting and comparator groups across these markers. A smaller number of studies [39,52] described greater reductions in HOMA-IR during fasting interventions. Data on insulin levels and insulin sensitivity markers are summarized in Table 1.

#### Results on PA monitoring and energy expenditure

PA was assessed or monitored in 12 studies, by accelerometers or questionnaires. Four studies included moderate or low-intensity exercise sessions [[52], [53], [54], [55], [56]], and 2 recommended daily walking [33,57]. However, only 2 trials included PA as a separate intervention arm [52,54], limiting conclusions regarding combined effects. More details on PA interventions and assessments are presented in Supplementary Тable 2.

None of the studies incorporating PA reported results on energy expenditure, including those with PA as a distinct intervention arm [52,54], as their primary outcomes focused predominantly on metabolic changes. Only 2 studies [34,59] reported PA energy expenditure, the first one comparing ADF with HD, and the second one comparing IF-P and CER. Both studies showed no changes in energy expenditure neither within nor between groups.

#### Results on functional performance and QoL

QoL outcomes were reported in only 1 study [49], and functional performance in 2 studies [52,55], highlighting major evidence gaps for patient-centered outcomes. Details appear in Supplementary Table 3. Where reported, no differences were observed between fasting and control groups, suggesting that fasting interventions did not adversely affect QoL or functional performance. Notably, 1 study [52] reported an increase in handgrip strength exclusively in the exercise and combined (fasting + PA) intervention groups when compared with the control group, indicating that fasting may confer additional functional benefits when combined with structured PA.

During the screening and data extraction phases, ongoing trials addressing the primary objective of the present review were identified. As results from these studies have not yet been published and therefore could not be included in the scoping review, details of ongoing trials without available results are provided in Supplementary Tables 4 to 6.

## Discussion

This scoping review mapped randomized trial evidence on IF and FF-related outcomes in middle-aged and older adults compared with HD or CER. To our knowledge, this is the first review assessing this outcome specifically in aging populations, as the only prior publication [60] focused exclusively on individuals without MS. The main finding is not that IF preserves skeletal muscle mass, but rather that the existing evidence base is limited, heterogeneous, and primarily based on short-term trials using FFM or lean mass as proxy outcomes.

Substantial variability was observed across study populations and methodologies. Several studies enrolled participants with overweight or obesity and with mean ages around 60 to 65 y, representing mixed middle-aged and older populations rather than geriatric cohorts. Studies specifically examining adults aged ≥70 y, who have a higher risk of developing sarcopenia [3], were limited. Consequently, findings observed in younger older adult cohorts may not be generalizable to more vulnerable geriatric populations.

In addition, IF protocols differed considerably in fasting duration, feeding windows, caloric restriction strategies, and integration with PA. Comparator groups and outcome reporting varied also substantially across studies, preventing robust characterization of intervention-specific patterns.

Across the included RCTs, IF led to generally small FFM changes, consistently with previous systematic reviews [5,7,[14], [15], [16]]. Nonetheless, greater reductions in FFM relative to HD [7,61] or CER [16,62] have also been reported. Importantly, the eligible studies provided only indirect and time-bounded information regarding muscle-related outcomes during IF interventions. FFM was assessed through DEXA or BIA, methods that assess overall body composition without a direct evaluation of skeletal muscle mass quantity and quality. Total FFM includes multiple nonfat tissues and does not adequately capture age-related functional decline or muscle-specific adaptations. Moreover, sarcopenia-related endpoints (e.g., ASMI, ALM or ALM/BMI), muscle strength and physical performance were rarely assessed. Thus, current evidence does not support the conclusion that IF in older adults preserves skeletal muscle mass in the long term or prevents muscle loss beyond the physiological decline associated with aging.

Potential mechanisms underlying the maintenance of FFM during IF may include compensatory increases in muscle protein synthesis during feeding periods, particularly when protein intake is concentrated within a shorter eating window. Among the 10 studies reporting protein intake, most studies found no differences between groups and heterogeneity in reporting protein intake (i.e., percentage of total energy intake compared with g/d) limited the ability to synthesize these findings. Moreover, only a few studies [41,42,55,56] reported data on actual eating windows adhered to by participants. Given the central role of nutrient intake timing in IF protocols, improved and standardized reporting of eating windows and dietary intake is strongly recommended in future trials.

Insulin also plays a key anabolic role in skeletal muscle, an effect that is attenuated under insulin-resistant conditions; thus, improvements in insulin sensitivity may contribute to FFM/lean mass proxy outcomes [63,64]. Similarly, metabolic flexibility, an essential component of adaptive energy metabolism, is impaired in insulin resistance [65]. Nevertheless, the regulation of insulin signaling, metabolic flexibility, and endocrine–anabolic responses remains poorly characterized in the context of aging and sarcopenia [[66], [67], [68]]. Most eligible studies reported no between-group differences in insulin-related outcomes; only 2 exceptions were observed [39,52], in 1 case likely due to the known effects of PA on insulin regulation. These findings are in contrast with previous systematic reviews in younger adults [69,70] or mixed-age populations [71,72].

Regarding secondary anthropometric outcomes, IF frequently reduced BW and FM, especially when compared with HD, as described in the literature [5,7,14,15,16]. It suggests that IF and CER may have comparable efficacy when total caloric intake is similar [71], although study heterogeneity precludes any firm conclusion. When considering the only eligible study combining protein pacing to IF, a small reduction in BW, FM and WC was observed, despite identical energy intake and expenditure among groups (58,59). Furthermore, 1 eligible RCT controlled for energy intake, nutrient quality and timing by having 4-arm design [49]. It showed that IF reduced BW and FM more than CER, but not when IF was prescribed in energy balance. Notably, substantial heterogeneity across study designs made it difficult to determine whether the benefits of IF were attributable to caloric restriction alone or reflected additional effects of fasting interventions.

Among analyzed fasting regimens, ADF and ADMF interventions frequently led to reductions in BW and FM, mostly when compared with HD [34,36,37,46,49] and, just in 1 case, with CER [49]. Although these findings are consistent with previous reviews [10,61], conclusions regarding comparative efficacy between fasting approaches in aging populations were precluded due to the scoping nature and the high heterogeneity in eligible studies. Literature in adults suggests that factors responsible for these potential differences between ADF and HD might be calorie intake and expenditure, and possibly nutrient quality. The biological rationale for ADF’s greater impact may relate to extended periods of metabolic switching and increased reliance on fat oxidation during fasting days [62]. However, its effects on energy expenditure, fat turnover, and long-term adherence remain unclear.

PA interventions were incorporated in only a subset of studies. Although the projects combining IF with PA [[52], [53], [54], [55], [56], [57]] reported reductions in FFM, FM, and BW, the limited number of studies and variability in training protocols prevented clear interpretation. Importantly, few trials assessed strength, mobility, balance, or physical performance, despite their relevance in aging populations. Thus, future research is needed, particularly given the importance of exercise in preventing and mitigating sarcopenia. Coupling a dietary intervention with aerobic and resistance training may also maximize health benefits by preserving muscle and bone mass, enhancing metabolic, cardiopulmonary, and cognitive function in older adults with obesity and diabetes [70,73,74].

Taken together, the current literature primarily supports descriptive observations regarding short-term body composition changes during IF interventions in middle-aged and older adults. The evidence does not currently support conclusions regarding long-term safety, preservation of skeletal muscle, prevention of sarcopenia, or healthy aging trajectories.

### Limitations

Despite rigorous adherence to methodological standards, several limitations should be acknowledged. First, substantial heterogeneity existed across studies in fasting protocols, caloric intake, intervention duration, participant characteristics, control conditions, and outcome assessment methods. This limits the comparability across trials.

Second, body composition was assessed using both BIA and DEXA, introducing methodological variability. Because BIA is strongly influenced by hydration status and hydration control procedures were rarely reported, some between-study differences in FFM may reflect measurement artifacts rather than true physiological changes. DEXA assessments may also vary by device model and calibration, which was not consistently reported. Most studies assessed exclusively total FFM, without using muscle-specific indices such as ASMI, ALM, or ALM/BMI, despite international recommendations [19,20,[27], [28], [29], [30]]. As a result, subtle or region-specific losses in skeletal muscle mass could not be evaluated, and the clinical implication of observed FFM changes remains uncertain.

Third, findings are mainly applicable to adults aged ∼45 to 65 y and to relatively healthy younger-old adults with overweight or obesity. They should not be extrapolated to frail older adults, adults aged ≥70 y, or individuals with established sarcopenia without additional evidence. Future studies should prespecify age strata, include sufficient numbers of adults aged ≥70 y, and evaluate whether fasting responses differ by age, baseline muscle status, sex, MS, protein intake, and PA.

Fourth, dietary intake, quality and distribution, and adherence to prescribed eating windows were insufficiently reported in many studies. Similarly, PA interventions varied widely in type, duration, and intensity, and were rarely standardized, constraining interpretation of combined effects.

Fifth, intervention duration was relatively short (typically ≤12 weeks). Given that age-related FFM decline develops gradually, longer-term RCTs are needed to evaluate the sustainability, safety, and clinical relevance of IF intervention in aging populations. Indeed, adherence in older adults seems to decline over time, and weight-loss maintenance beyond 6–12 months appears comparable to conventional CER [7,72,75,76]. In addition, the potential impact of IF on eating behaviors (e.g., leading to unhealthy food habits and obsessive eating-related mindsets) remains poorly understood [50,77].

Sixth, some potentially relevant trials employing alternative designs (e.g., pre–post or crossover) were not included, and no studies on Ramadan fasting met the eligibility criteria.

Finally, patient-centered outcomes such as QoL and functional performance were rarely assessed, despite their central importance in aging populations. Because functional decline, not body composition alone, drives disability in older adults, future studies should integrate patient-centered and performance-based outcomes.

In conclusion, this scoping review mapped randomized trial evidence on IF and FFM-related outcomes in middle-aged and older adults. The available evidence is heterogeneous and largely short-term, with most studies enrolling adults with overweight or obesity and using DEXA or BIA to assess total FFM or lean mass. Across the mapped studies, IF was commonly associated with reductions in BW and FM compared with HD, whereas FFM changes were generally small and inconsistently characterized. However, total FFM and lean mass are proxy outcomes and do not permit firm conclusions about skeletal muscle preservation, sarcopenia prevention, functional capacity, or long-term aging trajectories. Evidence remains particularly limited for adults aged ≥70 y, frail older adults, and individuals with established sarcopenia.

Furthermore, studies combining IF with PA interventions were relatively few and heterogeneous. Although some reported additional reductions in FM, the available data were insufficient to characterize consistent patterns regarding FFM or functional outcomes.

Future trials should include muscle-specific measures, ALM, strength and performance outcomes, dietary protein intake, PA assessment, longer follow-up, and age-stratified analyses to determine the relevance of IF for healthy aging.

## Author contributions

The authors’ responsibilities were as follows – SS, BB, LDR, HC: designed research; SS, BB: performed the review; MM: analyzed data; SS, BB, MM: interpreted the results; SS, BB, MM, JACG: wrote the paper; LDR, HC, JMO: edited and revised the manuscript and the Supplementary Materials; SS, BB: primary responsible for final content; and all authors: read and approved the final manuscript.

## Data availability

Data described in the manuscript are freely available without restriction as a supplemental spreadsheet and at https://osf.io/3y247.

## Declaration of Generative AI and AI-assisted Technologies in the Writing Process

The authors declare that no generative AI or AI-assisted technologies were used in the writing of this manuscript.

## Funding

This work has been funded by the R&D&I project PID2021-125899OB-I00, funded by MCIN/AEI/10.13039/501100011033/ and ERDF A way to build Europe. JACG has received funding from the Community of Madrid through a predoctoral grant for doctoral theses within the framework of the 2022 call for applications. The PhD scholarship of Sara Santero has been funded by the National Recovery and Resilience Plan, Mission 4 Component 2 Investment 1.3—Call for tender No. 341 of 15 March 2022 of Italian Ministry of University and Research funded by the European Union—Next Generation EU. Project code PE00000003, Concession Decree No. 1550 of 11 October 2022, adopted by the Italian Ministry of University and Research, CUP F13C22001210007, Project title “ON Foods—Research and innovation network on food and nutrition Sustainability, Safety and Security—Working ON Foods.” BB was funded by The Ministry of Science, Technological Development and Innovation of the Republic of Serbia, under Contract no. 451-03-137/2026-03/ 200117.

## Conflict of interest

JMO is an Editorial Board Member for Advances in Nutrition and played no role in the Journal’s evaluation of the manuscript. The other authors report no conflicts of interest.
