# Supplementary material for: Intermittent Fasting and Fat-Free Mass Outcomes in Middle-Aged and Older Adults: A Scoping Review
Source: Adv Nutr. 2026 Jul 2;17(7):100663. doi: 10.1016/j.advnut.2026.100663 (PMC13390093; doi:10.1016/j.advnut.2026.100663)
Supplement: Multimedia component 1 [file mmc1.pdf]

Intermittent fasting and fat-free mass outcomes in middle-aged and older adults: a scoping review

Santero et al.

Additional tables presented data extracted from the eligible studies

Supplementary Table 1. Study Implementation and Adherence

Supplementary Table 2. Physical Activity Component

Supplementary Table 3. Other secondary outcomes (quality of life and functional performance)

Supplementary Table 4. Characteristics of study protocols related to the research question of this scoping review for which results for our outcomes of interest have not been published yet.

Supplementary Table 5. Intervention and control characteristics, as well as planned study implementation and adherence, of study protocols related to the research question of this scoping review for which results for our outcomes of interest have not been published yet.

Supplementary table 6: Physical Activity Component of study protocols related to the research question of this scoping review for which results for our outcomes of interest have not been published yet.

Supplementary Table 1: Study Implementation and Adherence. Drop-out reasons were classified as follows: 1) adherence-related, divided in 1a) lack of motivation or interest in following the dietary guidelines derived from randomization results, ii) schedule-related: difficulty in attending the visits due to transport, distance, lack of time, work related problems, including also loss of contacts; 2) medical reasons (e.g., unrelated disease, surgical procedures, pregnancy etc); 3) other reasons (not clearly reported in the paper). Abbreviations: ADF, alternate day fasting; CER, caloric energy restriction; CONT, control; IF, intermittent fasting; INT, intervention; NA, not assessed; ns, not significant; s, significant; TRE, time restricted eating.

First Author (Year)	Baseline number of participants (INT; CONT)	Number of participants who completed the study (INT; CONT)	Dropout Rate (%)	Drop-out Reasons	Visit Frequency	Diet Adherence Method	Diet adherence rate (%)	Actual eating timing followed by participants (INT; CONT)	Dietary Intake Assessment Method
Teng NI (2013) (ref. 32)	56 participants (28 in INT; 28 in CONT)	56 participants (28 in INT; 28 in CONT)	0%	/	0, 6 weeks, 12 weeks; weekly telephone calls	fasting log book and food diaries	100% of INT to caloric restriction; 92.9% to 2 days of fasting	NA	Diet History Questionnaire (baseline) and 3-days food diary (0, 6, 12w), analysed through food software
Panizza CE (2019) (ref. 33)	60 participants (30 in INT; 30 in DASH)	54 participants (26 in INT; 28 in DASH)	10%	4 adherence-related (1 lack of motivation or interest; 3 schedule-related); 2 medical reasons	0, 12 weeks; almost weekly telephone calls	self-reported how many successful days	90.6%	NA	mobile App to capture images before and after meals (baseline, 5-6, 11w); not specified how it was analysed
Stekovic S. (2019); Tripolt N.J. (2018) (ref. 34,35)	60 participants (30 in INT, 30 in CONT)	57 participants (29 in INT, 28 in CONT)	5%	3 adherence-related (lack of motivation or interest)	week 0, 3, 4	continuous glucose monitoring	NA	NA (strict adherence requirements, thus the participants actually followed the research protocol)	semi-quantitative FFQ (week 0, week 3), analysing caloric intake with a German nutritive value database

Varady KA (2013)<br>(ref. 36)	32 participants (16 in INT, 16 in CONT)	30 participants (15 in INT, 15 in CONT)	6.25%	2 adherence-related (1 lack of motivation or interest, 1 schedule-related)	weekly	food records	98% ± 5%	NA (98 ± 5% adherence at baseline to the fast day protocol in the ADF group, with ns changes over the course of the study)	food log for extra foods, weighed leftovers food items. 3-days food record (week 1, week 12) analysed through food software
Obermayer A (2023); Obermayer A (2022)<br>(ref. 37,38)	46 participants (22 in INT; 24 in CONT)	44 participants (20 in INT; 24 in CONT)	4.3%	1 adherence-related (schedule-related);<br>1 medical reasons	every 4 weeks; telephone contacts in between	food diary	> 75% adherence for completers	NA (91% of the participants achieved >75% adherence to the fasting protocol)	NA
Cienfuegos S. (2020)<br>(ref. 39)	58 (19 in 4-h TRE, 20 in 6-h TRE, 19 in CONT)	49 (16 in 4-h TRE, 19 in 6-h TRE, 14 in CONT)	15.5%	8 adherence-related (schedule-related);<br>1 medical reasons	weekly	daily self-reported through diet adherence log for eating window	NA	Baseline: ns difference between the 4-h TRE (10.8 ± 0.5 h) and 6-h TRE (10.3 ± 0.6 h) group<br>During the INT: self-reported adherence of 6.2 ± 0.2 d/week for 4-h TRE (eating window: 3:00–7:00 pm daily) and of 6.2 ± 0.1 d/week for 6-h TRE (eating window: 1:00–7:00 pm daily) with ns changes over the course of the trial<br>Not reported the eating window of the CONT	7-days food record (baseline, 8 weeks), analysed with dedicated food program
Domaszewski P. (2023)<br>(ref. 40)	116 (61 in TRE, 55 in CONT)	108 (54 in TRE, 54 in CONT)	6.9%	7 adherence-related (lack of motivation or interest);<br>1 medical reasons	weekly	daily self-reported by marking the days on which they completed the diet plan	> 98%	NA (data were excluded from the analysis if the number of days without the recommended TRE plan exceeded 10 %; adherence > 98%)	NA

Parr EB (2024)<br>(ref. 41)	51 participants (26 in INT, 25 in CONT)	43 participants (22 in INT, 21 in CONT)	15.7%	6 adherence-related (3 not specified, 2 lack of motivation or interest, 1 schedule-related);<br>2 medical reasons	visit at 0, 1, 2, 4 months	self-reported adherence questionnaire	≥ 75%	<u>Baseline</u>: ns differences between groups in eating window duration (11.0–11.7 h ca.; from 08:30-9:00 a.m. to 08:30-09:00 p.m.)<br><u>During the INT</u>: s changes in eating window behaviours in the TRE group, i.e. reduction in their eating window by -2.8 h ca. (95%CI: -3.5 to -2.1 h), delaying their first eating occasion by 1.6 h ca (95%CI: +2.2 to +1.0 h) and having an earlier last eating occasion by -1.2 h ca. (95%CI: -0.8 to -1.7 h), across the 6-months.<br>Ns differences in the CONT group	2-weeks diet records through mobile App with photos (baseline, 1, 2, 4 months); not specified how it was analysed
Dote-Montero M (2025); Dote-Montero M (2024)<br>(ref. 42,43)	197 participants (49 in eTRE; 52 in lTRE; 47 in ssTRE; 49 in CONT)	183 participants (47 in eTRE; 48 in lTRE; 42 in ssTRE; 46 in CONT)	7.6%	3 adherence-related (2 lack of motivation or interest, 1 schedule-related);<br>5 medical reasons;<br>6 other reasons	every 2 weeks	daily food record	eTRE: 85.3%; (95% CI, 80.6 - 90.0); lTRE: 88.1%; (95% CI, 84.9 - 91.3); ssTRE: 85.4%; (95% CI, 81.3 - 89.5)	<u>During the INT</u>: s reduction in the eating window in the eTRE group (median: 7.7 h; interquartile range, 0.5; median 09:45 a.m. to 5:30 p.m.), lTRE group (median: 7.4 h; interquartile range; median 0.8; median 2:20 to 9:30 p.m.) and ssTRE group* (median: 7.6 h; interquartile range, 0.4; median 12:20 to 8:00 p.m.) than in the CONT group (median: 13.4 h; interquartile range, 1.2; median 08:30 to 22:00)<br>* 14% of participants chose an eTRE schedule (before	24-h recalls (0, 12w), analysed through food software

								10:00 a.m.) and 43% a lTRE (after 1:00 p.m.)<br>Not specified for CONT	
He CJ (2021)<br>(ref. 44)	205 participants (102 in INT; 103 in CER)	173 participants (88 in INT; 85 in CER)	15.6%	24 adherence-related (6 not specified, 10 lack of motivation or interest, 8 schedule-related);<br>1 medical reasons;<br>7 other reasons	monthly	food diary	100% [inability to adhere to the dietary protocol was an exclusion criteria]	NA	questionnaire; not further specified
Kunduraci Y (2020)<br>(ref. 45)	70 participants (35 in INT, 35 in CER)	65 participants (32 in INT; 33 in CER)	7.1%	3 adherence-related (lack of motivation or interest);<br>2 medical reasons	visits at 0, 4 w, 8 w and 12 weeks; weekly phone contacts	food diaries and fasting logbooks	100% [3 participants with a low adherence rate were considered as drop outs]	NA (each participant followed different 16-h fasting windows, such as 04.00–08.00 a.m., 05.00 p.m.–09.00 a.m., 06.00 p.m.–10.00 a.m., or 07.00 p.m.–11.00 a.m. fasting hours)	24-h recall (baseline, weeks 4, 8, 12), analysed through food software
Teong X.T. (2023); Teong X.T. (2021); Teong X.T. (2020)<br>(ref.46,47,48)	209 participants (85 in TRE, 83 in CER, 41 in CONT)	163 participants at 6 months (69 in TRE, 62 in CER, 32 in CONT)	22%	41 adherence-related (29 lack of interest or motivation, 12 schedule-related);<br>4 medical reasons;<br>1 other reasons	fortnightly	7-day food records	NA	NA	diet diary smartphone application, analysed using food software

Hutchison A.T. (2019); Teong X.T. (2021)<br>(ref. 49,50)	88 participants (25 in IF70, 25 in IF100, 26 in CER, 12 in CONT)	79 participants (22 in IF70; 22 in IF100; 24 in CER; 11 in CONT)	10.2%	5 adherence-related (3 lack of motivation or interest, 2 schedule-related);<br>3 medical reasons;<br>1 other reasons	weekly	daily checklists	NA	NA	calculated from dietary records through food software
Pavlou V (2023)<br>(ref. 51)	75 participants (25 in TRE, 25 in CER, 25 in CONT)	69 participants (23 in TRE; 22 in CER; 24 in CONT)	8%	4 adherence-related (1 lack of motivation or interest, 3 schedule-related);<br>1 medical reasons;<br>1 other reasons	weekly up to 3 months, then biweekly	dietary log (the CONT group used an App)	NA	NA (self-reported adherence of 6.1 ± 0.8 days per week; s reduction in adherence in INT over the course of the study; ns changes for CER and CONT)	automated 24-h recall; not specified how it was analysed
Keawtep P (2024); Keawtep P (2023)<br>(ref. 52,53)	92 participants (23 in DIET INT; 23 in EX INT; 23 in COMB; 23 in CONT)	80 participants (21 in DIET INT; 19 in EX INT; 20 in COMB; 20 in CONT)	13%	9 adherence-related (schedule-related);<br>3 medical reasons	NA	food diary through mobile App	88.83% for the DIET INT; 87.30% for COMB	NA (mean self-reported adherence to ADF was 88.83%, and 87.30% for the combined group)	NA
Bhutani S (2013)<br>(ref. 54)	83 participants (18 in COMB, 25 in ADF, 24 in PA, 16 in CONT)	64 participants (16 in COMB,	22.9%	18 adherence-related<br>(4 lack of motivation or	weekly	extra food log	ADF: 80 ± 9%; COMB: 81 ± 7% to	NA (81 ± 7% adherence in the COMB and 80 ± 9% in ADF, calculated as number of fasting day adherent/number	NA

		16 in ADF, 16 in PA, 16 in CONT)		interest, 14 schedule-related)<br>1 medical reasons			fasting days	of fasting days in the week x 100)	
Kotarsky, C.J. (2021)<br>(ref. 55)	23 participants (13 in INT, 10 in CONT)	21 participants (11 in INT, 10 in CONT)	8.7%	2 medical reasons	after 4 weeks	3-day dietary records	NA	<u>During the INT</u>: on average between 12:00 p.m. and 7:30 p.m. for TRE (7.0 ± 0.8 h; required between 12:00 p.m. and 8:00 p.m.), and between 8:00 a.m. and 8:30 p.m. (11.9 ± 1.4 h) for CONT<br>More than one day of noncompliance would exclude the participant from the study	3-day dietary records (baseline, week 1, 4 and 7), analysed through food software
Lin YJ (2022)<br>(ref. 56)	63 women (30 in INT; 33 in CER)	63 women (30 in INT; 33 in CER)	0%	/	na but daily contacts	record of daily food intake, including pictures	84%	<u>During the INT</u>: s reduction in the eating window for TRE (from 11.5 ± 1.0 h to 8.6 ± 1.6 h; 8-h time period recommended, either h 10:00 a.m.-6:00 p.m. or 12:00 -20:00 p.m.)<br>Not specified for CONT	food intake record (baseline, 8 weeks); not specified how it was analysed
Carter S (2018)<br>(ref. 57)	137 participants (70 in INT; 67 in CER)	97 participants (51 in INT; 46 in CER)	29.2%	22 adherence-related (3 not specified, 9 lack of motivation or interest, 10 schedule-related);<br>7 medical reasons;<br>11 other reasons	every 2 weeks in the first 3 months, then every 2 to 3 months	Blood glucose control, weight, diet checklists, scales to weigh foods	97% at 3 months in IF; 44% at 12 months in IF	NA	NA

Arciero et al. 2022, Arciero et al. 2023 (ref .58, 59)	41 participants (21 in INT; 20 in CONT)	39 participants (20 in INT; 19 in CONT)	4.9%	2 adherence-related non-compliant with the meal plan	Measurements in weeks 0, 5, 9; weekly dietitian meetings	2-day dietary records	95.2%	NA	food intake records (for 2 days during CON (week 0), week 4, and week 8) analysed through food software

Supplementary Table 2: Physical Activity Component. Abbreviations: ADF, alternate day fasting; CER, caloric energy restriction; CONT, control; INT, intervention; IPAQ, International Physical Activity Questionnaire; NA, not assessed; PA, physical activity; TRE, time restricted eating.

First Author (Year)	Type of PA intervention	Intensity	Sessions/Week & Duration	Monitoring Techniques	PA Monitoring Results
Teng NI (2013) (ref. 32)	none, PA level was not evaluated and not monitored	NA	NA	no	NA
Panizza CE (2019) (ref. 33)	none, PA was evaluated by the questionnaire and all participants were recommended to walk	low	a walk up to one hour per day, up to five days a week.	no	NA
Stekovic S. (2019); Tripolt N.J. (2018) (ref. 34,35)	none, PA level was evaluated (IPAQ) and monitored	NA	NA	accelerometer (for 7 days at baseline and at 4 weeks)	Non-significant change neither pre- and post-treatment nor between groups comparisons; **activity energy expenditure (kcal/Week):** median (IQR) INT change from baseline: -211 (-1,057 to 805) CONT change from baseline: -365 (-942.3 to 329.8)
Varady KA (2013)	none, PA level was not assessed	NA	NA	no	NA

(ref. 36)					
Obermayer A (2023); Obermayer A (2022)<br>(ref. 37,38)	none, PA level was evaluated (IPAQ)	NA	NA	no	NA
Cienfuegos S. (2020)<br>(ref. 39)	none, participants were asked to maintain their usual PA level	NA	NA	accelerometer (for 7 days at baseline and at 8 weeks)	difference (4-h TRE): -597 ± 702 steps/day; difference (6-h TRE): 53 ± 457 steps/day; difference (CONT): 359 ± 533
Domaszewski P. (2023)<br>(ref. 40)	none, participants were asked to maintain their usual PA level	NA	NA	no	NA
Parr EB (2024)<br>(ref. 41)	none, PA was monitored	NA	NA	accelerometer (for 14 days at baseline, 1, 2, 4, 6 months)	Difference (INT vs CONT) at 6w: –482±7422 steps/da
Dote-Montero M (2025); Dote-Montero M (2024)<br>(ref. 42,43)	none, PA level was evaluated (IPAQ)	NA	NA	accelerometer (for 14 days at baseline and 10 weeks)	NA
He CJ (2021)<br>(ref. 44)	none, participants were asked to maintain their usual PA level	NA	NA	no	NA
Kunduraci Y (2020)<br>(ref. 45)	none, PA level was evaluated (IPAQ)	NA	NA	no	NA
Teong X.T. (2023); Teong X.T. (2021);	none, participants were asked to maintain their usual PA level	NA	NA	accelerometer (for at least 7 days at baseline, 6, 18 months)	difference (iTRE): -211 [-873, 513] steps/day in iTRE<br>difference (CER): +362 [-311, 1100] in CER

Teong X.T. (2020)<br>(ref.46,47,48)					difference (SC): -183 [-1200, 985] in SC
Hutchison A.T. (2019); Teong X.T. (2021)<br>(ref. 49,50)	none, participants were asked to maintain their usual PA level	NA	NA	no	NA
Pavlou V (2023)<br>(ref. 51)	none, participants were asked to maintain their usual PA level	/	/	accelerometer (for 7 days at 0, 12, 24 weeks)	Difference (TRE vs CONT) at 6w: 1020 ± 5360<br>(CER vs CONT) at 6w: 422 ± 7647<br>(TRE vs CER) at 6 w: 598 ± 7770
Keawtep P (2024); Keawtep P (2023)<br>(ref. 52,53)	yes, aerobic and anaerobic training, and simultaneous physical-cognitive sessions in PA group and COMB group	moderate	physical-cognitive program for 60 min/day, 3 days/week for 3 months; not specified duration and frequency of training types	PA record (logbook or App)	NA
Bhutani S (2013)<br>(ref. 54)	yes, combined and exercise groups participated in the exercise intervention.	moderate	three times per week under supervised conditions, for 12 weeks. Exercise was performed using stationary bikes and elliptical machines. Training duration and intensity increased incrementally during the study, from 25-min duration at an intensity of 60% HR max in weeks 1-4 , to a 40-min duration at an intensity of 75% HRmax in weeks 10-12	Exercise compliance was assessed by<br>recording attendance at each supervised exercise session	NA
Kotarsky, C.J. (2021)<br>(ref. 55)	yes, resistance training (3 different workouts) and aerobic training (treadmill or equivalent) in both INT and CONT	moderate or vigorous	resistance training: non-consecutive days for 8 weeks (3 sets of 12 repetitions with no more than 60 s of rest between exercises and sets; total duration not specified)	record of training sessions attended; accelerometer (for at least 4 days at 2, 5, 8w)	T2w - INT: 8116 ± 828 steps/day; 7541 ± 782 in CONT<br>T8w - INT: 7929 ± 835 steps/day; 8039 ± 686 in CONT

			aerobic training: 50-60 min/day to total 300 min of moderate, or 150-min of vigorous physical activity per week		
Lin YJ (2022)<br>(ref. 56)	yes, in both groups but not further specified	low	eight 30-min exercise sessions, once per week	no	NA
Carter S (2018)<br>(ref. 57)	yes, aerobic training (walking) in both groups	low	all participants were asked to increase their step count by 2000 and maintain this increase over the length of the trial.	accelerometer (for 2 weeks at baseline, 2 weeks)	T0: 6800 (3187) steps/day in INT; 5889 (2893) in CER. T12 months: na
Arciero et al. 2022, Arciero et al. 2023 (ref .58, 59)	None, the inclusion criteria was that participants were either sedentary or lightly active (<30 minutes, 2 d/wk of organized physical activity)	NA	NA; participants were asked to maintain their physical activity lifestyle	ActiGraphs were worn for for 2 days during weeks 0, 4, and 8.	Non-significant change neither pre- and post-treatment nor between groups comparisons;<br>Physical activity energy expenditure (PAEE, kcal/day):<br>IF-P: Pre: 287 ± 41 / Post: 346 ± 45,<br>CER: Pre: 376 ± 50 / Post: 350 ± 53<br>IF *vs* CER [MD (95%CI)]: 85 (−51.2, 221.2)

Supplementary Table 3. Other secondary outcomes (quality of life and functional performance). Abbreviations: EQ-5D-5L, EuroQol 5 dimensions 5 levels questionnaire; SF36, Rand Short Form Health Survey with 36 questions; QoL, quality of life.

First Author (Year)	QoL assessment method	QoL results	**Functional performance** (e.g., SPPB, gait speed, time-up-and-go test, 400 m walk, 5-time chair stand test, 6-m walk)	Functional performance results
Stekovic S. (2019); Tripolt N.J. (2018)<br>(ref. 34,35)	SF-36 (visit 1, visit 3, follow-up)	methodology was described in study protocol, but results were not published yet	yes, HANDGRIP (visit 1, visit 3, follow-up). Isometric grip strength using a handgrip dynamometer (JAMAR, Nottinghamshire, UK).	methodology was described in study protocol, but results were not published yet
Obermayer A (2023); Obermayer A (2022)<br>(ref. 37,38)	EuroQol-5D	A significant difference in the change of perceived health (EuroQol-5D visual analog scale) between the fasting (from 70 ± 20 to 74 ± 21) and control group	NA	NA

		(from 70 ± 20 to 65 ± 23) was observed ($P = 0.043$).		
Dote-Montero M (2025); Dote-Montero M (2024)<br>(ref. 42,43)	EQ-5D-5L and SF-36	methodology was described in study protocol, but results were not published yet; by the response of authors, publication is under review process	NA	NA
Teong X.T. (2023); Teong X.T. (2021); Teong X.T. (2020)<br>(ref.46,47,48)	SF-36 (week 0, 8, 24, 76)	methodology was described in study protocol, but results were not published yet	yes, HANDGRIP STREGTH: digital hand-dynamometer (Jamar®Plus +, Patterson Medical, Cedarburg, WI,USA) at weeks 0, 8, 24, and 76.	methodology was described in study protocol, but results were not published yet
Hutchison A.T. (2019); Teong X.T. (2021)<br>(ref. 49,50)	SF-36 (week 0, 8 weeks)	There were no statistical differences between groups in the change in markers of QOL	NA	NA
Pavlou V (2023)<br>(ref. 51)	Appetite, sleep, mood, quality of life, readiness for change, eating disorder symptoms - assessed by validated questionnaires at week 0, 12, 24. (not specified which questionnaires)	methodology was described in study protocol, but results were not published yet.	NA	NA
Keawtep P (2024); Keawtep P (2023)<br>(ref. 52,53)	NA	NA	Physical fitness was measured using 6-minute walk test (6MWT), muscle strength was measured using hand grip strength test for the upper extremity strength, and 30-second Chair Stand Test (30-s CST) for the lower extremity strength	No results reported for the 6MWT.<br>Only the exercise and combined intervention groups showed greater improvements in hand grip strength when compared with the control group ($p < 0.05$).

				Nevertheless, there was no significant difference in predicted maximal oxygen consumption and grip strength among combined, diet, and exercise groups (p > 0.05). No statistically significant difference between groups for chair stand test.
Kotarsky C.J. (2021)<br>(ref. 55)	NA	NA	1) HANDGRIP: Jamar Hydraulic Hand Dynamometer (Sammons Preston Rolyan).<br>2) a 3-min step test was conducted according to the protocol designed by the YMCA (American College of Sports Medicine, 2017).<br>3) A Biodex Pro4 system dynamometer (Biodex Medical Systems) was used to measure lower body muscle strength and endurance of the right leg.	1) there were no statistical differences compared to baseline, neither for fasting, nor for the control arm.<br>2&3) no significant knee extension strength peak torque, knee extension endurance total work, plantar flexion strength peak torque, dorsiflexion strength peak torque, plantar flexion endurance total work and dorsiflexion endurance total work

Supplementary Table 4. Characteristics of study protocols related to the research question of this scoping review for which results for our outcomes of interest have not been published yet. Abbreviations used: BMI, body mass index; CONT, control; INT, intervention; NA, not addressed; RCT, randomized controlled trial; TRE, time restricted eating.

First Author (Year) [NCT]; trial name	Study Design	Country	Study Setting	Eligible population (age range)	Cardiometabolic disease	Intervention	Type of Fasting (eating window)	Control
Lee, D.J. (2024) [NCT05565638]<br>PROlonged nightly	RCT (2 parallel arms, 1:1)	USA	hospital, community	≥18 years old	overweight or obesity (BMI of ≥25 kg/m^2)	TRE (4 months, 14-hour<br>nighttime fasting	14-hour<br>nighttime	healthy lifestyle education

FASTing (PROFAST)						intervention)	fasting	
Schübel R. (2018); Schübel R (2016); Pannen ST (2021)<br>[NCT02449148]; HELENA Trial	RCT (3 parallel arms, 1:1:1)	Germany	1 hospital and 1 research center (Heidelberg University Hospital and the German Cancer Research Center)	35–65 years old	overweight participants, BMI ≥ 25 kg/m2 and <40 kg/m^2; hypertension	12 weeks duration; intermittent calorie restriction (ICR): 5:2 diet, 5 d without energy<br>restriction and 2 d with 75% energy deficit, net weekly energy<br>deficit ~20% (n = 49)	5:2 diet	CONT1:<br>continuous calorie restriction (CCR): , daily energy deficit ~20%, (n = 49)<br>CONT2:<br>no advice to restrict energy (n = 52)
El-Outa A (2021) [NA]<br>iFast	RCT (4 parallel arms, 1:1:1:1)	Lebanon	university, community	18–64 years old	no	duration 12 weeks; 8h TRE,<br>INT1: Fasting + PA,<br>INT2: Fasting-no PA	16 h fasting, 8 h ad libitum eating, day period not specified	CONT1: non-fasting + PA<br>CONT2: non-fasting - no PA
Rizvi ZA (2024) [NCT05521945]	RCT (3 parallel arms, 1:1:1)	Pakistan	community	40-60 years old	BMI > 25 kg/m^2;<br>elevated fasting blood glucose,<br>hypertension,<br>hypercholesterolemia, hypertriglyceridemia	12 weeks duration,<br>8 h TRE, without caloric restriction	16 h fasting, 8 h ad libitum eating, from 1 pm to 9 pm	CONT1: No intervention, regular normal diet, encouraged not to change their eating regimen or their<br>usual lifestyle.<br>CONT2: customised diet, primarily based on<br>carbohydrate, protein and fat intake, adjusted to participants' BMI.
Gabel K (2022) [NCT05114798]<br>TRE compared with	RCT (3 parallel arms),<br>12-month	USA	community	45-65 years old	obesity (BMI of 30 to 49.99 kg/m^2) and increased risk for colorectal cancer,	6 months duration, 8 h TRE, without caloric restriction	ad libitum eating interval: 12 pm - 8 pm	continuous caloric restriction, 25% energy restriction daily

daily calorie restriction for weight loss and colorectal cancer<br>risk reduction trial (TRE-CRC trial)	(6-month intervention, 6-month maintenance)				prediabetes			
[NCT03457870]<br>ChANgE	RCT (4 parallel arms)	UK	NA	≥ 60 years old; older adults with overweight or obesity (BMI 25-35 kg/m^2)	NA	INT1: 5:2 fasting with caloric restriction; INT2: chewing plus 5:2 fasting with caloric restriction (asked to chew 1 piece of gum for 10' 3 times/day)	2 consecutive days of a very low calorie diet, 5 days of normal healthy eating for 3 months	continued habitual eating behaviour
[NCT05880095]<br>ENSATI	RCT (3 parallel arms)	Spain	communit y	55-70 years	not specified, but Type 1 diabetes and<br>Type 2 diabetes with poor glucose control were exclusion criteria	Mediterranean diet with TRE.<br>Participants will be allowed to consume water and non-caloric drinks during the fasting period.	10 h eating window of participant's choice between 6 am to 8 pm.<br>Participants will be advised to follow the 10h TRE during weekdays and weekends.	CONT1: unrestricted Mediterranean diet. Neither caloric restriction nor time-eating restriction will be indicated. CONT2: Mediterranean diet with 25% energy restriction.
[NCT05865639]<br>Time Restricted Fasting and Aerobic Exercise Interventions	RCT (4 parallel arms)	China	NA	45-69 years old;<br>Sedentary time ≥8 hours	not specified, but parameters (BMI, blood triglycerides, HDL cholesterol) will be assessed during	8 weeks intervention;<br>INT1: TRE<br>INT2: TRE + physical activity	eating window maximum 8 h; not specified at	CONT1: Aerobic exercise<br>CONT2: Aerobic exercise & Time

Among Sedentary Adults				per day	study	(PA)	which part of the day	Restricted Eating<br><br>CONT3: no intervention
[NCT05424042]<br><br>The health, aging, and later-life outcomes (HALLO-P)	RCT (3 parallel arms)	USA	communit y	≥ 60 years old adults with overweight or obesity	indication for weight loss (e.g., hypertension, hyperlipidemia, elevated waist girth, controlled diabetes)	9 months duration, 8 h TRE, without caloric restriction	consumption of all daily caloric intake within an 8-hour window of time, with no restrictions on caloric intake	CER, 20% reduction in caloric intake; CONT1: In-Person Caloric Restriction Arm; CONT2: Remote Caloric Restriction Arm
[NCT05593939]<br><br>Slow Age: Interventions to Slow Aging in Humans	RCT (3 parallel arms)	Denmark	NA	≥ 65 years old, in good general health	no, inclusion criteria is good general health	12 weeks duration,<br><br>8 h TRE, without caloric restriction	8 h ad libitum eating, self-selected eating window	CONT: No intervention arm

Supplementary Table 5. Intervention and control characteristics, as well as planned study implementation and adherence, of study protocols related to the research question of this scoping review for which results for our outcomes of interest have not been published yet.

Abbreviations: BIA, bioelectrical impedance analysis; CONT, control; DEXA, dual energy x-ray absorptiometry; INT, intervention; MR, magnetic resonance imaging; NA, not addressed; TRE, time restricted eating.

**First Author (Year)**<br><br>**[NCT]**	**Diet Composition; beverages allowed during fasting**	**Nutritional Guidelines/Pro tocol Referenced**	**Follow-u p Period**	**Method for Muscle Mass Assessment technique**	**Visit Frequency**	**Diet Adherence Method**	**Dietary Intake Assessment Method**

Lee, D. J. (2024) [NCT05565638]	not specified; plain coffee, plain tea, zero-calorie sodas, and calorie-free sweeteners	NA	no	DEXA	one-on-one telephone counseling by a health coach and automated SMS text messaging to support fasting goals	Feasibility is assessed as participant retention (percent dropout in each arm) and percentage of days participants achieved a ≥14-hour fast.	24-hour dietary recalls by questionnaires to assess food and beverage consumption (ideally for 1 weekday and 1 weekend day)
Schübel R. (2018); Schübel R (2016); Pannen ST (2021) [NCT02449148]	ICR: Meals on the energy-restricted days are based on a list with preselected food items sorted by different food groups. Participants are asked to choose four food items out of the vegetable group, two out of the low-fat dairy product group and one food item out of each the meat/fish, carbohydrate and fruit group. CER: substitution of energy-dense foods	German Nutrition Society	Intervention: 12 weeks; Maintenance: following 12 weeks (24 weeks in total) and following 26 weeks (50 weeks in total)	MR	at the baseline and at the end of each period (intervention - week 12, maintenance - week 24, follow up - week 50)	questionnaire on adherence to dietary intervention and a fasting diary. The nutritionist takes into account the self-reported diet of the individual participant, as stated in the 7-day food record	7-day food record and pictured food portions - calculations of energy and nutrient intake by PRODI 6.3 (Nutri-Science GmbH, Hausach, Germany), a software based on the German Nutrient Data Base including intakes on all seven days of the week.
El-Outa A (2021) [NA]	not specified; during the fasting hours, participants should drink water and they can also drink non-caloric beverages such as	/	no	BIA	monthly visits and 24-h recalls every 2 weeks	quantitative blood ketone measurement at each visit will enable quantification of the ketosis state induced by intermittent fasting; this will also serve as an indicator of adherence to intermittent fasting in the respective	the 24-h recall collected 5 times over the study period in order to ensure consistency in dietary intake (3 times during week-days and 2 times during the weekend)

	sugar-free black coffee and tea					group	
Rizvi ZA (2024) [NCT05521945]	participant's regular dietary plan during 8 h of non-fasting intervals, i.e., 1 pm to 9 pm, with few instructions. Participants were advised to stay adequately hydrated with simple water, black coffee, black tea, or lemonade water with some fibre twice a day and to avoid carbonated and sugary beverages altogether.	/	no	not measured, only BMI and waist circumference were measured	at baseline and at the end of the trial	combination of weekly phone calls and messages throughout the trial duration	not specified
Gabel K (2022) [NCT05114798]	not be required to monitor calories or restrict types or quantities of foods. Water and calorie free beverages (black coffee, tea, diet soda, etc.) will be permitted during the fasting period. Only a total of two diet sodas will be permitted each day	/	6 months maintenance (10-h TRE, ad libitum eating window: 10 am to 8 pm)	dual energy x-ray absorptiometry (DEXA)	once a week	Each day, participants will respond to a text message to indicate their eating start and stop time	by 7-day food records; Participants will be asked to measure the portion amounts of foods consumed with household measures and to record the timing of food intake. Food

[NCT03457870] (ChANgE)	NA	NA	no	NA	NA	NA	NA
[NCT05880095] (ENSATI)	Mediterranean Diet	MEDAS (Mediterranean Diet Adherence Screener) criteria	interventio n for 6 months, and follow-up after 6 months (at 1 year)	DEXA	3 months	Daily time of the eating window (hours) and the daily fasting period (hours) will be assessed through questionnaires to record the meal time every day	Food Questionnaires and MEDAS (Mediterranean Diet Adherence Screener)
[NCT05865639] Time Restricted Fasting and Aerobic Exercise Interventions Among Sedentary Adults	NA	NA	no	NA	NA	Self-reported by WeChat application, but only for exercise sessions	Self-reported by using the Adult Eating Behavior Questionnaire (AEBQ) and by Mint Health application
[NCT05424042] The health, aging, and later-life outcomes (HALLO-P)	not specified; TRE arm is allowed ad libitum food intake during 8 h eating window	NA	no	DEXA	monthly for the first 6 months, and group settings, During the remaining 3 months there will be one group and one individual meeting each month	log into the study-specific Companion App each day to document the beginning and end of their cycles with timing of meals/snacks. TRE sustainability will be assessed and met if at least 75% of participants eating within the window on >80% of days.	Data on self-reported energy intake will be collected and nutrients and food groups analyzed using the publicly available National Cancer Institute's Automated Self-Administered 24-Hour (ASA24) dietary assessment tool.
[NCT05593939] Slow Age: Interventions to Slow Aging in	not specified; TRE arm is allowed ad libitum food intake during 8 h eating window	NA	no	BIA	NA	NA	NA

Humans							

Supplementary table 6: Physical Activity Component of study protocols related to the research question of this scoping review for which results for our outcomes of interest have not been published yet. Intensity is classified as high, moderate or low. Abbreviations: NA, not addressed.

First Author (Year) [NCT]	Type of PA intervention	Intensity	Sessions/Week & Duration	Monitoring Techniques
Lee, D. J. (2024) [NCT05565638]	none, but physical activity levels were assessed	/	/	accelerometers for 7 days at the baseline
Schübel R. (2018); Schübel R (2016); Pannen ST (2021) [NCT02449148]	none, but physical activity levels were assessed	/	/	accelerometers for 7 days at the baseline and during the last week of the intervention.
El-Outa A (2021) [NA]	resistance training exercise - The participant will be introduced to an intermediate-intensity calisthenics exercise routine of two exercises which will consist of push-ups (regression with knees on the ground is optional) and body-weight chairseated squats (progression to air squats is optional) - participants will be asked to adhere to the form of exercise they start with	intermediate-intensity calisthenics exercise routine of two exercises (push-ups and body-weight chairseated squats)	3 times a week	the participant will record the number of repetitions and the duration taken to perform the exercises)
Rizvi ZA (2024) [NCT05521945]	none, but physical activity levels were assessed	/	/	International Physical Activity Questionnaire (IPAQ)

Gabel K (2022) [NCT05114798]	instructed to maintain their<br>current level of physical activity throughout the duration of the trial	/	/	accelerometers for 7 days
[NCT03457870]<br>(ChANgE)	no intervention	NA	NA	NA
[NCT05880095]<br>(ENSATI)	no intervention	NA	NA	NA
[NCT05865639]<br>Time Restricted Fasting and Aerobic Exercise Interventions Among Sedentary Adults	Exercise sessions with aerobic exercise. Intervention period will be eight weeks.	Each session lasts for 60-65 minutes, each period consists of 5 min warm up, 45 min formal training and 5-10 min relaxation training.	3 times a week	Measured by using accelerometer for 7 consecutive days
[NCT05424042]<br>The health, aging, and later-life outcomes (HALLO-P)	none, but physical activity levels were assessed	/	/	Participants will use wrist-worn Fitbit step monitors to track their physical activity and receive feedback via the app, with a goal to promote movement across the day, continuously increasing their daily step count.
[NCT05593939]<br>Slow Age: Interventions to Slow Aging in Humans	One study arm was an exercise arm, with aerobic exercise as the intervention.	Moderate to vigorous intensities i.e., ~60-90% heart rate (HR) max.; Aerobic exercise will be performed in agreement with guidelines for exercise in older adults from the American College of Sports Medicine.	12 weeks, with a frequency of 5 days/week, totaling 150-300 min/week.	None, but aerobic exercise sessions are in person, which ensures monitoring of participants.
