# Supplementary material for: Intermittent Fasting and Fat-Free Mass Outcomes in Middle-Aged and Older Adults: A Scoping Review
Source: Adv Nutr. 2026 Jul 2;17(7):100663. doi: 10.1016/j.advnut.2026.100663 (PMC13390093; doi:10.1016/j.advnut.2026.100663)
Supplement: Multimedia component 2 [file mmc2.pdf]

Intermittent fasting and fat-free mass outcomes in middle-aged and older adults: a scoping review

Santero et al.

Meta-analyses on Changes in Anthropometric Parameters

Methods

Exploratory meta-analyses were conducted using R software version 4.1.2 and the metafor package. Although formal meta-analysis is not performed in scoping reviews, quantitative synthesis was incorporated **exploratorily** to enhance data interpretability where data were sufficiently homogeneous. These analyses were not intended to generate definitive pooled effects but to provide preliminary quantitative insights (hypothesis-generating) to guide future systematic reviews. These results should be taken with caution given the high heterogeneity, the absence of a formal risk-of-bias assessment and the limited consideration of key muscle-related confounders (e.g., protein intake and structured exercise).

Effect sizes were expressed as mean differences (MD) in changes from baseline between intervention and control groups, with corresponding 95% confidence intervals. When required and when data permitted, standard deviations were derived using established formulas. A random-effects model was applied due to anticipated heterogeneity arising from differences in populations, fasting regimens, caloric intake, and measurement techniques.

Analyses were performed separately for comparisons with habitual diet (HD) and with Continuous Energy Restriction (CER). Sensitivity analyses were stratified by fasting type and metabolic syndrome status. Only outcomes for which adequate data were available were included in each model.

Results

Meta-analyses were conducted separately for studies comparing fasting with habitual diet (HD) and fasting with continuous energy restriction (CER). Given the heterogeneity in intervention designs, populations, and measurement methods, the absence of a formal risk-of-bias assessment and the limited consideration of key muscle-related confounders, the analyses should be interpreted as **exploratory**. Furthermore, the high heterogeneity (i.e. I^2 values exceeding 99% are reported across multiple analyses) undermines the interpretability of pooled estimates, even in exploratory analyses, suggesting that these results reflect direction of effect rather than magnitude.

Stratification by intervention and control group (HD or CER)

Effects of Fasting on Changes in Fat-Free Mass

Figure S1 presents changes in FFM across fasting interventions compared with habitual diet. The analysis included 12 studies with 17 fasting intervention arms (471 fasting vs. 285 control participants). Although FFM loss was greater in 12 fasting arms, the pooled mean difference was small and not statistically significant (MD = -0.36 kg; 95% CI: -0.75 to 0.04). Heterogeneity was substantial (I^2 = 99.9%, $p < 0.001$), reflecting differences in fasting protocols, measurement methods (DEXA vs BIA), and caloric intake. Despite statistical heterogeneity, the magnitude of change was small and unlikely to be clinically relevant in relation to sarcopenia risk.

Figure S1: Effect of fasting *vs*. habitual diet (HD) on changes in **fat-free mass** (in kg), stratified by fasting interventions.

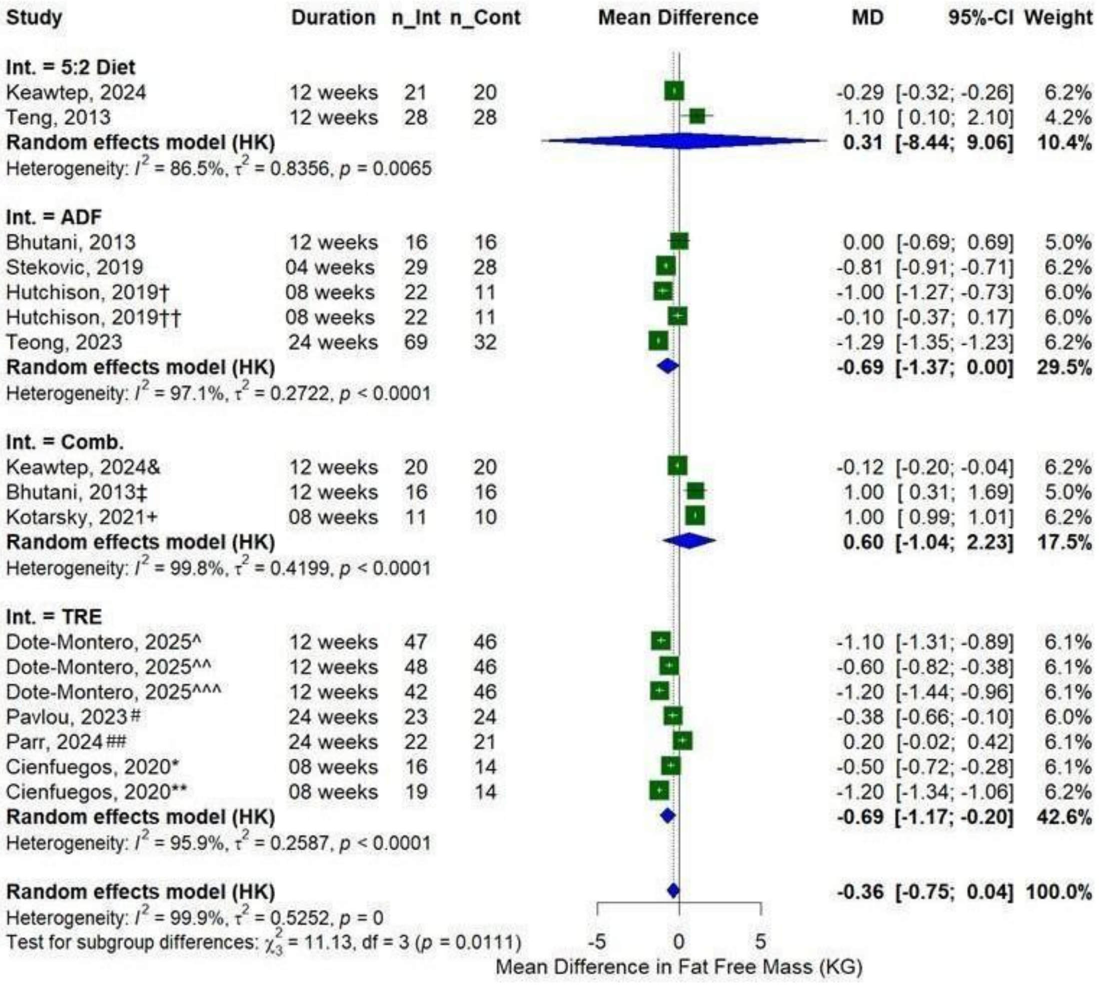

Mean difference indicates the mean difference of change from fasting intervention *vs*. control group. Each horizontal line represents the 95% confidence interval (CI) for an individual study. The green squares indicate the effect estimates from individual studies, with the square size proportional to the study weight. The blue diamond represents the pooled effect estimate, with its width showing the 95% CI.

Marks: †IF70; ††IF100; &5:2 Diet+physical activity; ‡mADF+physical activity; +8hTRE+ physical activity; ^eTRE; ^^ iTRE; ^^^ssTRE; *4h TRE; **6h TRE; #8h TRE; ##9h TRE.

Abbreviations: TRE – time restricted eating; eTRE – early time restricted eating, iTRE – intermediate time restricted eating, ssTRE – self-selected time restricting eating; ADF – alternate day fasting; CI – confidence interval; Cont – control group; Int – intervention group; MD – mean difference; n – number of participants in each arm.

Figure S2 shows FFM changes comparing fasting with CER (9 studies; 367 fasting vs. 335 CER participants). FFM loss was modestly greater in 7 fasting arms, but the pooled effect was not statistically significant (MD = -0.29 kg; 95% CI: -0.68 to 0.10). Heterogeneity was again high ($I^2 = 99.4\%$, $p < 0.001$). Given the similarity in caloric intake between fasting (especially ADMF/5:2) and CER arms in several trials, comparable effects on FFM were expected.

Figure S2. Effect of fasting *vs*. Continuous Energy Restriction (CER) on changes in **fat-free mass** (in kg), stratified by fasting intervention.

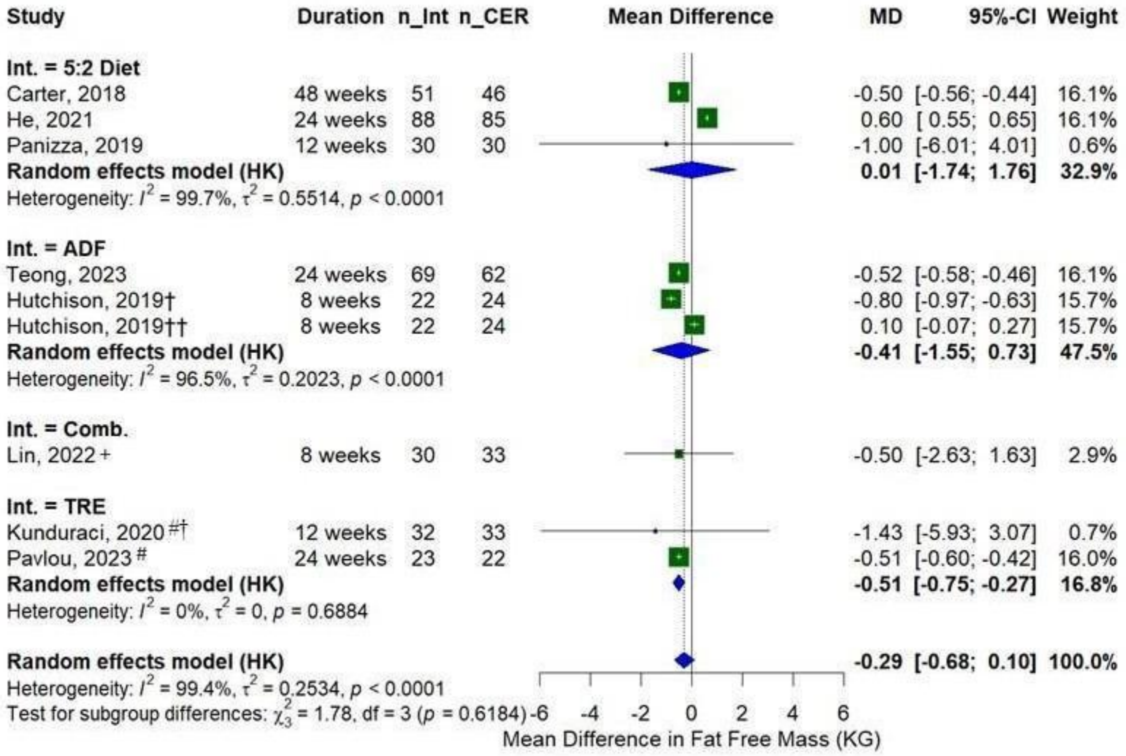

Mean difference indicates the mean difference of change from fasting intervention *vs*. control group. Each horizontal line represents the 95% confidence interval (CI) for an individual study. The green squares indicate the effect estimates from individual studies, with the square size proportional to the study weight. The blue diamond represents the pooled effect estimate, with its width showing the 95% CI.

Marks: †IF70; ††IF100; +8hTRE+ physical activity; #8h TRE.

Abbreviations: TRE – time restricted eating; ADF – alternate day fasting; CI – confidence interval; CER – caloric energy restriction group; Int – intervention group; MD – mean difference; n – number of participants in each arm.

Effects of Fasting on Changes in Fat Mass

Figure S3 shows changes in fat mass compared with habitual diet. Thirteen studies (20 intervention arms; 508 fasting vs. 324 control participants) demonstrated a significant pooled reduction in fat mass with fasting (MD = -2.41 kg; 95% CI: -3.02 to -1.81). Reductions were greatest in interventions combining fasting with physical activity, followed by ADF protocols. These findings align with known metabolic effects of fasting patterns that alternate low and high nutrient availability.

Figure S3: Effect of fasting *vs*. habitual diet (HD) on changes in **fat mass** (in kg), by fasting intervention.

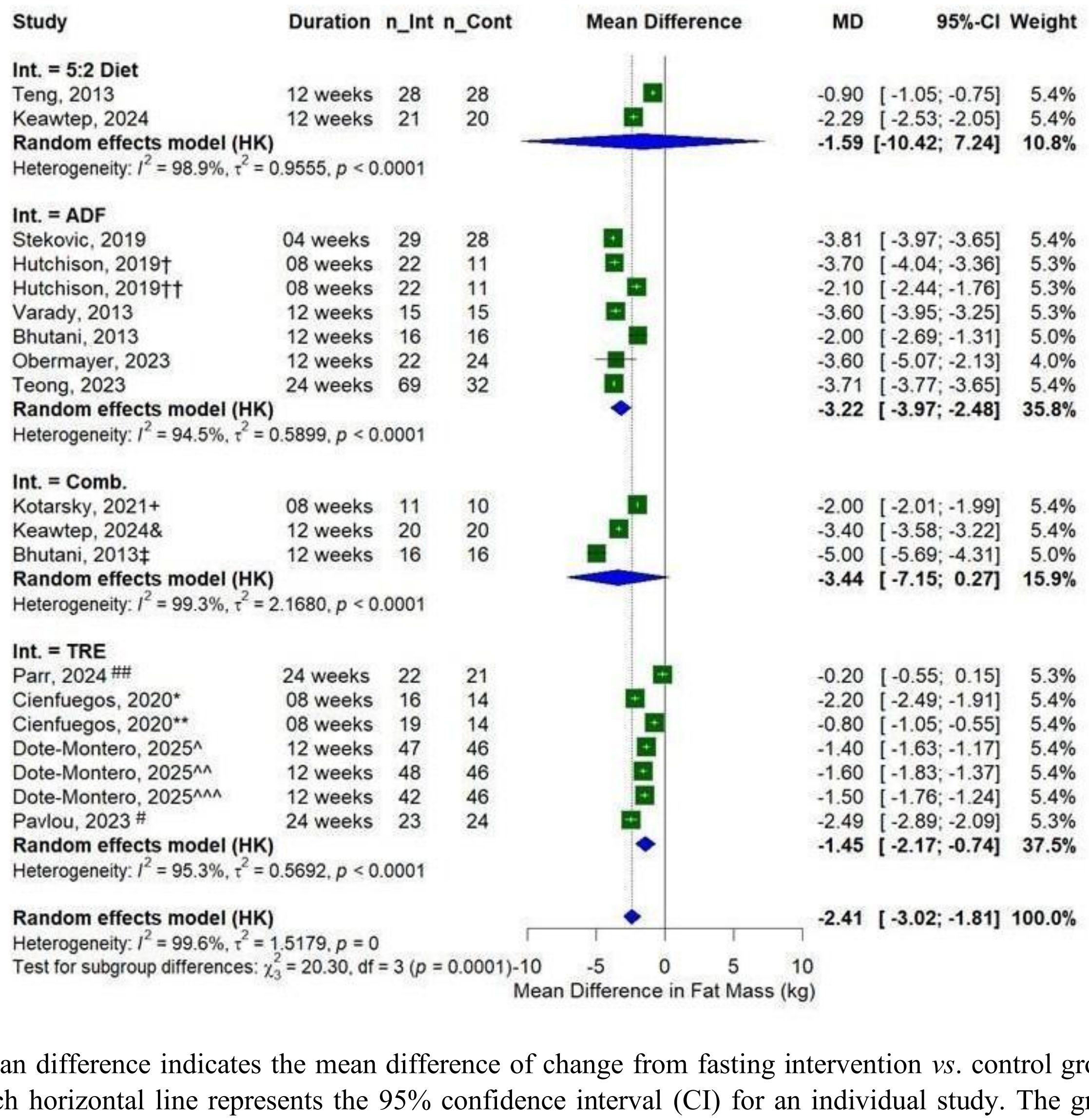

Mean difference indicates the mean difference of change from fasting intervention *vs*. control group. Each horizontal line represents the 95% confidence interval (CI) for an individual study. The green squares indicate the effect estimates from individual studies, with the square size proportional to the study weight. The blue diamond represents the pooled effect estimate, with its width showing the 95% CI. The blue diamond at the base of the plot demonstrates the pooled effect estimates and confidence intervals from all RCTs included.

Marks: †IF70; ††IF100; &5:2 Diet+physical activity; ‡mADF+physical activity; +8hTRE+ physical activity; ^eTRE; ^^ iTRE; ^^^ssTRE; *4h TRE; **6h TRE; #8h TRE; ##9h TRE.

Abbreviations: TRE – time restricted eating; eTRE – early time restricted eating, iTRE – intermediate time restricted eating, ssTRE – self-selected time restricting eating; ADF – alternate day fasting; CI – confidence interval; Cont – control group; Int – intervention group; MD – mean difference; n – number of participants in each arm.

Figure S4 compares fasting with CER (8 studies; 337 fasting vs. 301 CER). Fasting resulted in a modest but statistically significant additional reduction in fat mass (MD = -0.68 kg; 95% CI: -1.21 to -0.15). Although small in magnitude, this difference suggests possible metabolic or behavioral advantages of fasting regimens over daily caloric restriction. Combined fasting + physical activity arms showed slightly larger reductions, although the small number of studies limits interpretation.

Figure S4: Effect of fasting *vs*. Continuous Energy Restriction (CER) on changes in **fat mass** (in kg), by fasting intervention.

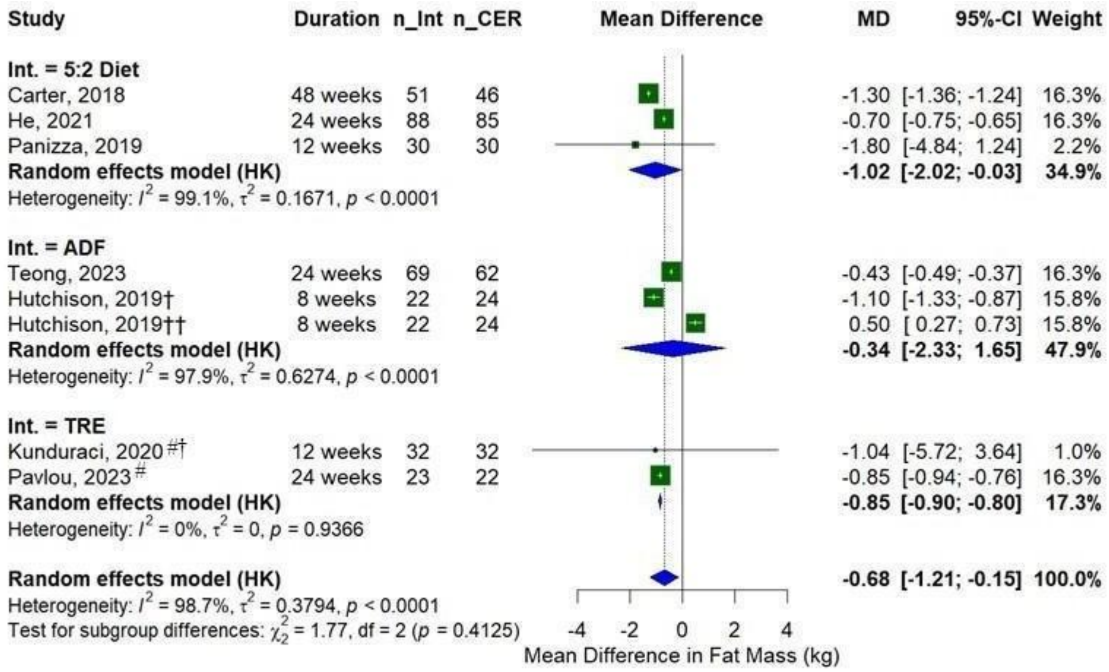

Mean difference indicates the mean difference of change from fasting intervention *vs*. CER. Each horizontal line represents the 95% confidence interval (CI) for an individual study. The green squares indicate the effect estimates from individual studies, with the square size proportional to the study weight. The blue diamond represents the pooled effect estimate, with its width showing the 95% CI. The blue diamond at the base of the plot demonstrates the pooled effect estimates and confidence intervals from all RCTs included.

Marks: †IF70; ††IF100; #8h TRE.

Abbreviations: TRE – time restricted eating; ADF – alternate day fasting; CI – confidence interval; CER – caloric energy restriction group; Int – intervention group; MD – mean difference; n – number of participants in each arm.

Effects of Fasting on Changes in Body Weight

Sixteen studies (404 fasting vs. 243 control participants) compared fasting with habitual diet (Figure S5). All fasting interventions produced significantly greater weight loss than habitual diet (pooled MD = -3.49 kg; 95% CI: -4.26 to -2.72). Heterogeneity was high (I^2 = 99.4%, p < 0.001), likely due to differences in fasting type, adherence, trial duration, and participant metabolic profiles. Among fasting protocols, ADF produced the largest weight loss, followed by fasting combined with physical activity, the 5:2 diet, and TRE.

Figure S5: Effect of fasting *vs*. habitual diet (HD) on changes in body weight (in kg), by fasting intervention.

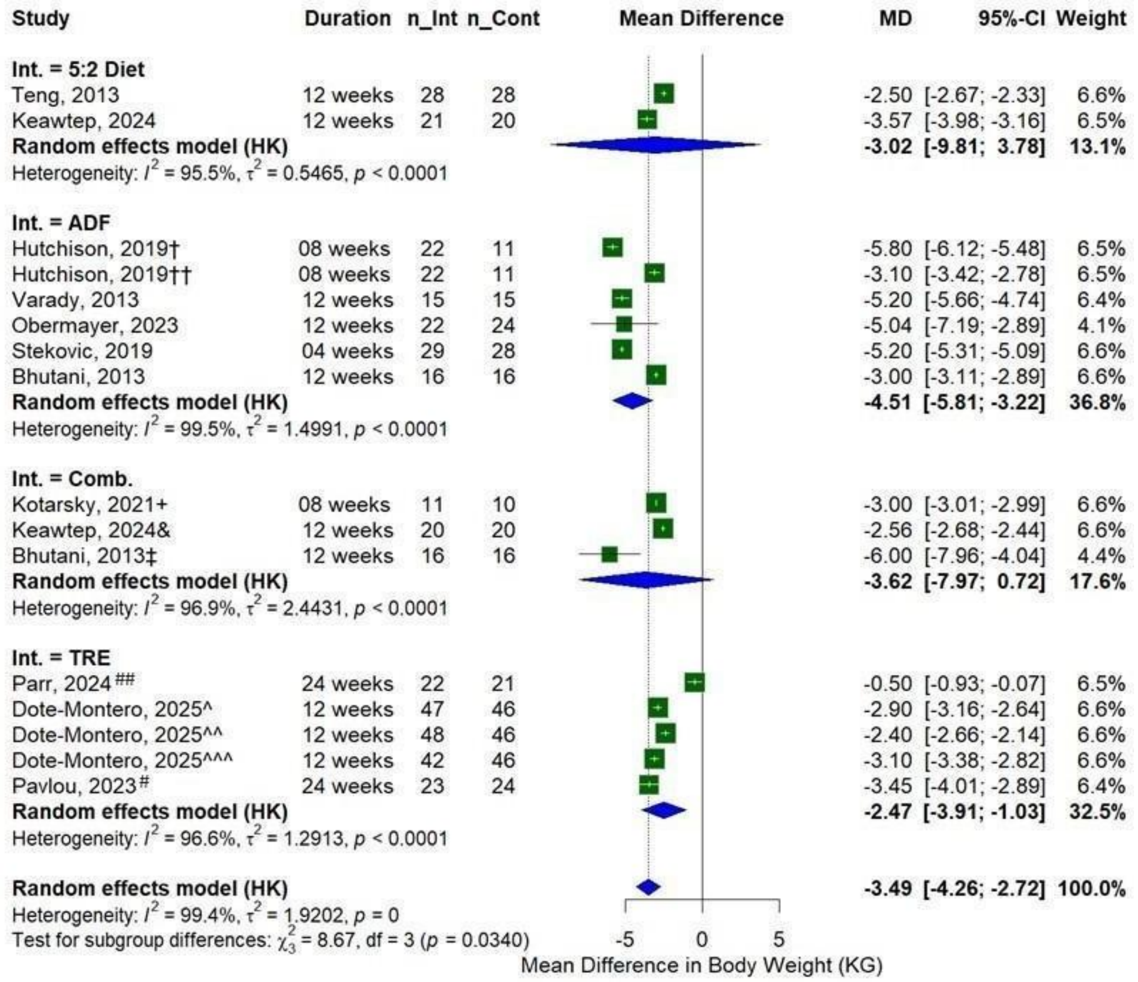

Mean difference indicates the mean difference of change from fasting intervention *vs*. control group. Each horizontal line represents the 95% confidence interval (CI) for an individual study. The green squares indicate the effect estimates from individual studies, with the square size proportional to the study weight. The blue diamond represents the pooled effect estimate, with its width showing the 95% CI. The blue diamond at the base of the plot demonstrates the pooled effect estimates and confidence intervals from all RCTs included.

Marks: †IF70; ††IF100; &5:2 Diet+physical activity; ‡mADF+physical activity; +8hTRE+ physical activity; ^eTRE; ^^ iTRE; ^^^ssTRE; #8h TRE; ##9h TRE.

Abbreviations: TRE – time restricted eating; eTRE – early time restricted eating, iTRE – intermediate time restricted eating, ssTRE – self-selected time restricting eating; ADF – alternate day fasting; CI – confidence interval; Cont – control group; Int – intervention group; MD – mean difference; n – number of participants in each arm; MS – metabolic syndrome.

Figure S6 summarizes fasting vs CER (9 studies; 367 fasting vs. 335 CER). Eight of nine studies showed greater weight loss in fasting groups; however, the pooled effect did not reach statistical significance (MD = -0.83 kg; 95% CI: -1.74 to 0.08). Given similar caloric restriction across many fasting and CER arms, these findings indicate broadly comparable efficacy for weight reduction.

Figure S6: Effect of fasting *vs*. Continuous Energy Restriction (CER) on changes in body weight (in kg), by fasting intervention.

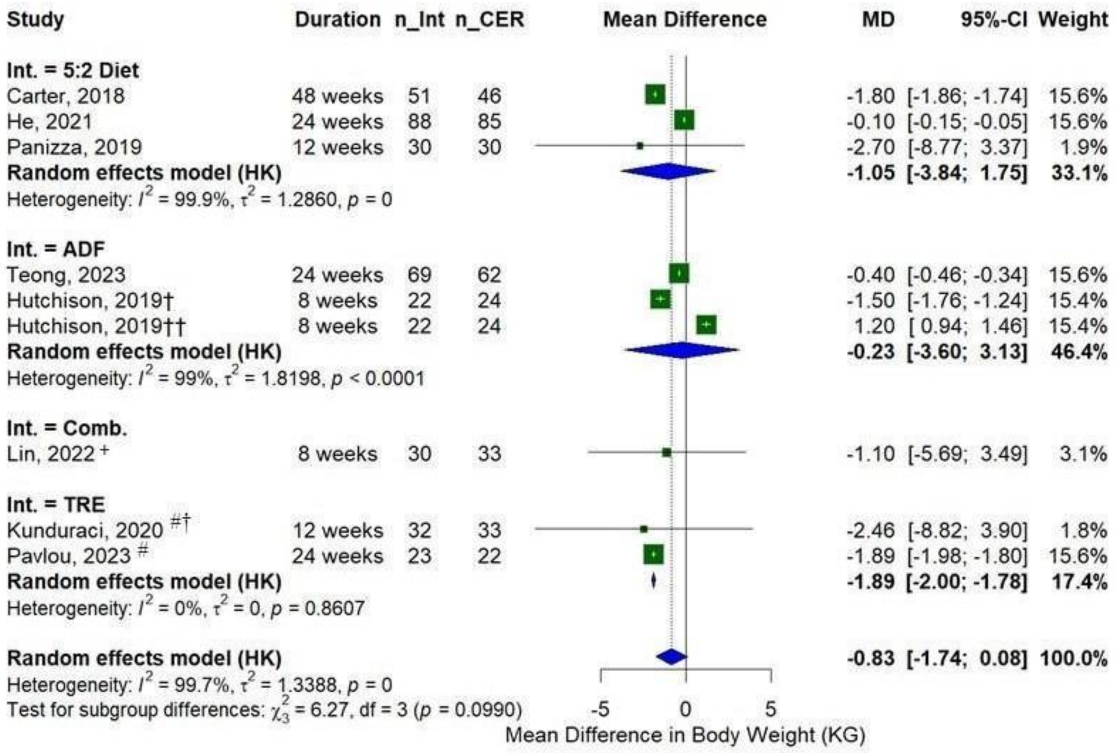

Mean difference indicates the mean difference of change from fasting intervention *vs*. control group. Each horizontal line represents the 95% confidence interval (CI) for an individual study. The green squares indicate the effect estimates from individual studies, with the square size proportional to the study weight. The blue diamond represents the pooled effect estimate, with its width showing the 95% CI. The blue diamond at the base of the plot demonstrates the pooled effect estimates and confidence intervals from all RCTs included.

Marks: [†]IF70; [††]IF100; +8hTRE+ physical activity; [#]8h TRE.

Abbreviations: TRE – time restricted eating; ADF – alternate day fasting; CI – confidence interval; CER – caloric energy restriction group; Int – intervention group; MD – mean difference; n – number of participants in each arm.

Effects of Fasting on Changes in Visceral Fat Mass and Waist Circumference

Figures S7 and S8 present analyses for visceral fat and waist circumference. Compared with habitual diet, fasting consistently reduced visceral fat (pooled MD = -0.10 kg; 95% CI: -0.15

to -0.06) and waist circumference (pooled MD = -4.36 cm; 95% CI: -5.98 to -2.74). These reductions occurred across all fasting types and were not meaningfully modified by MS status. When compared with CER, differences in visceral fat and waist circumference were small and not statistically significant, suggesting fasting and CER have comparable effects on central adiposity when calories are similarly restricted.

Figure S7. Effect of fasting *vs*. control (A-habitual diet as a control, B-CER as a control) on changes in **visceral fat mass** (in kg), stratified by fasting intervention.

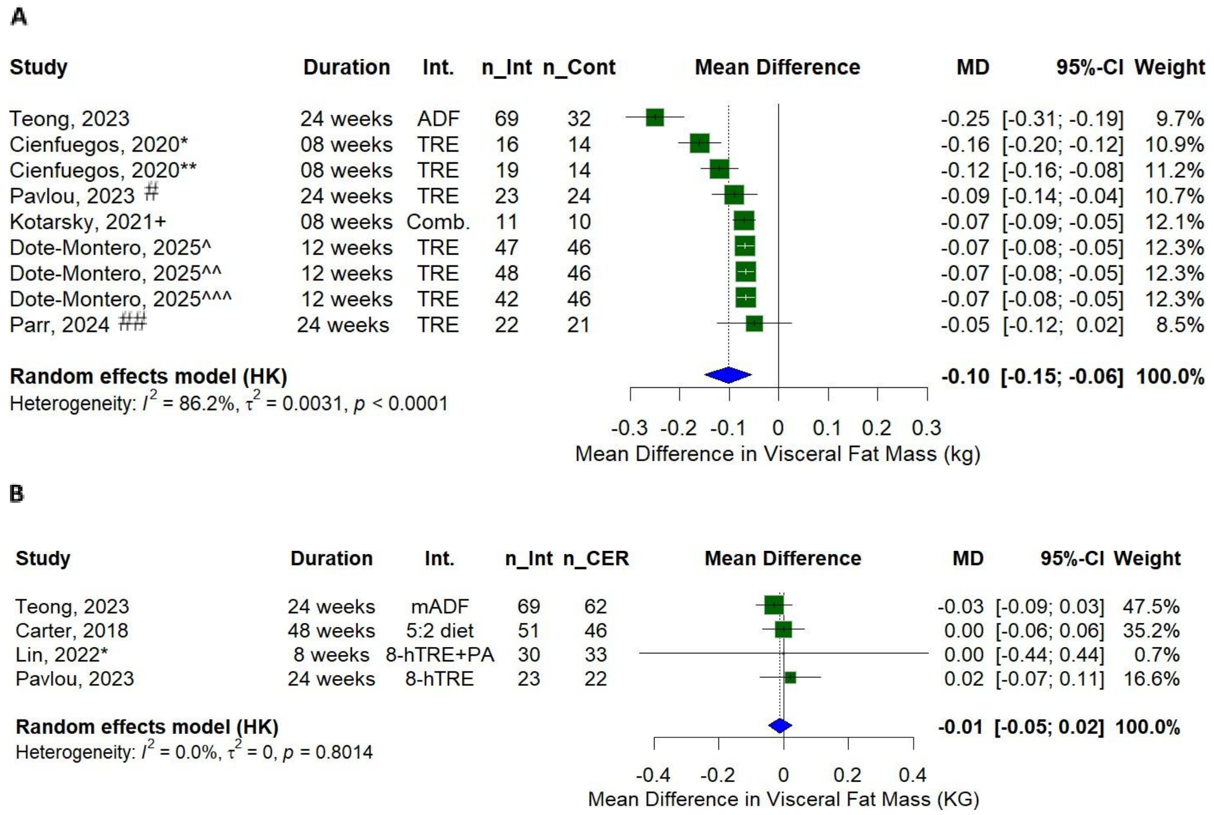

Mean difference indicates the mean difference of change from fasting intervention *vs*. control group. Each horizontal line represents the 95% confidence interval (CI) for an individual study. The green squares indicate the effect estimates from individual studies, with the square size proportional to the study weight. The blue diamond represents the pooled effect estimate, with its width showing the 95% CI. The blue diamond at the base of the plot demonstrates the pooled effect estimates and confidence intervals from all RCTs included.

Marks: *4h TRE; **6h TRE; #8h TRE; ##9h TRE; +8h TRE+physical activity; ^8h eTRE; ^^8h iTRE; ^^^8h ssTRE.

Abbreviations: TRE – time restricted eating; eTRE – early time restricted eating, iTRE – intermediate time restricted eating, ssTRE – self-selected time restricting eating; ADF – alternate day fasting; CI – confidence interval; Cont – control group; Int – intervention group; MD – mean difference; n – number of participants in each arm.

Figure S8. Effect of fasting *vs.* control (A-habitual diet as a control, B-CER as a control) on changes in **waist circumference** (in cm), stratified by fasting intervention.

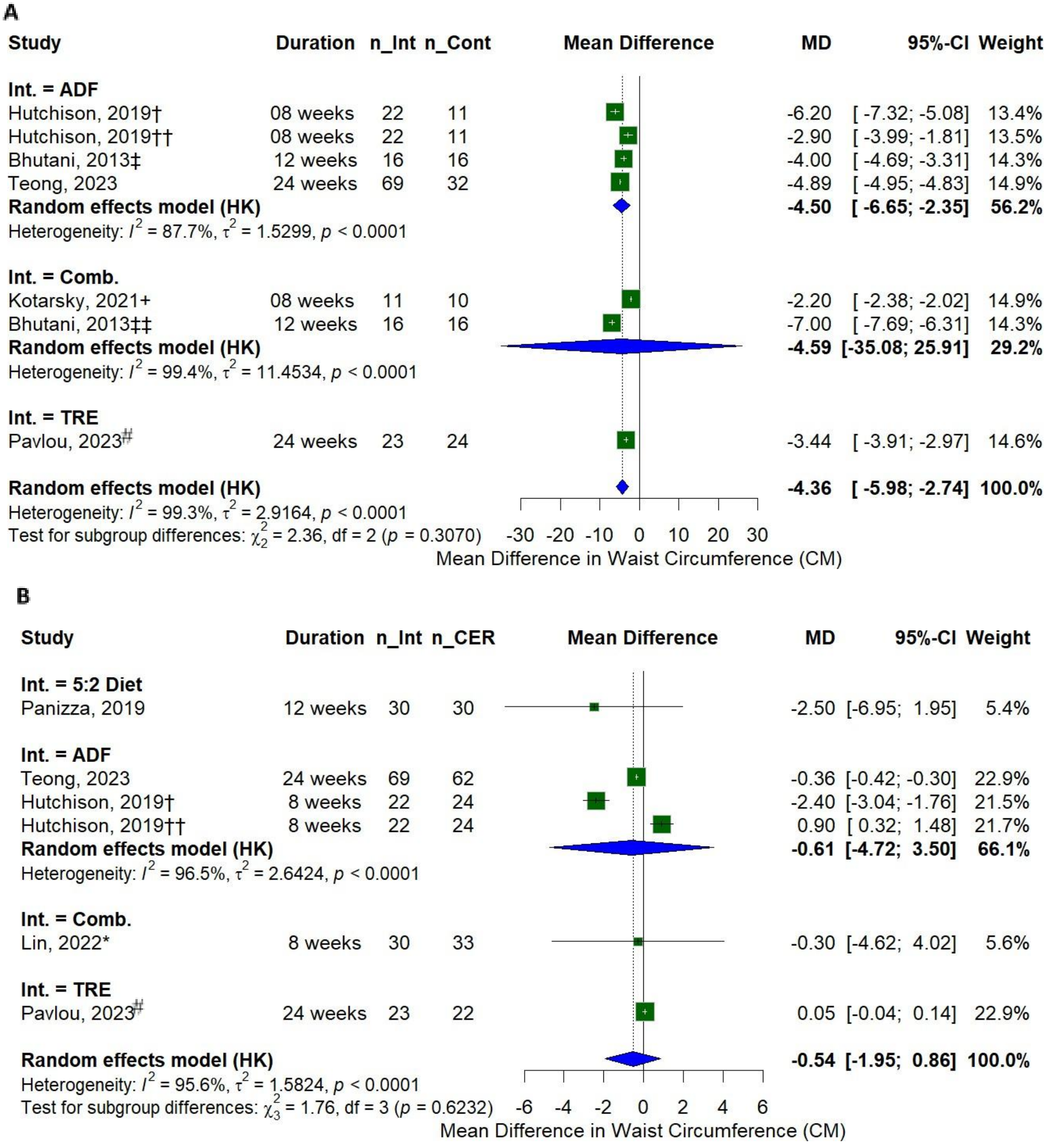

Effect of fasting *vs.* control on changes in waist circumference (in cm), by fasting intervention. Mean difference indicates the mean difference of change from fasting intervention *vs.* control group. Each horizontal line represents the 95% confidence interval (CI) for an individual study. The green squares indicate the effect estimates from individual studies, with the square size proportional to the study weight. The blue diamond represents the pooled effect estimate, with its width showing the 95% CI. The blue diamond at the base of the plot demonstrates the pooled effect estimates and confidence intervals from all RCTs included.

Marks: †IF70; ††IF100; &5:2 Diet+physical activity; +8h TRE+physical activity; ‡mADF+physical activity; #8h TRE.

Abbreviations: TRE – time restricted eating; ADF – alternate day fasting; IF - intermittent fasting; CI – confidence interval; Cont – control group; Int – intervention group; MD – mean difference; n – number of participants in each arm.

Stratification by metabolic syndrome

Figure S9. Effect of fasting *vs*. control (A-habitual diet as a control, B-CER as a control) on changes in **fat-free mass** (in kg), stratified by presence of metabolic syndrome.

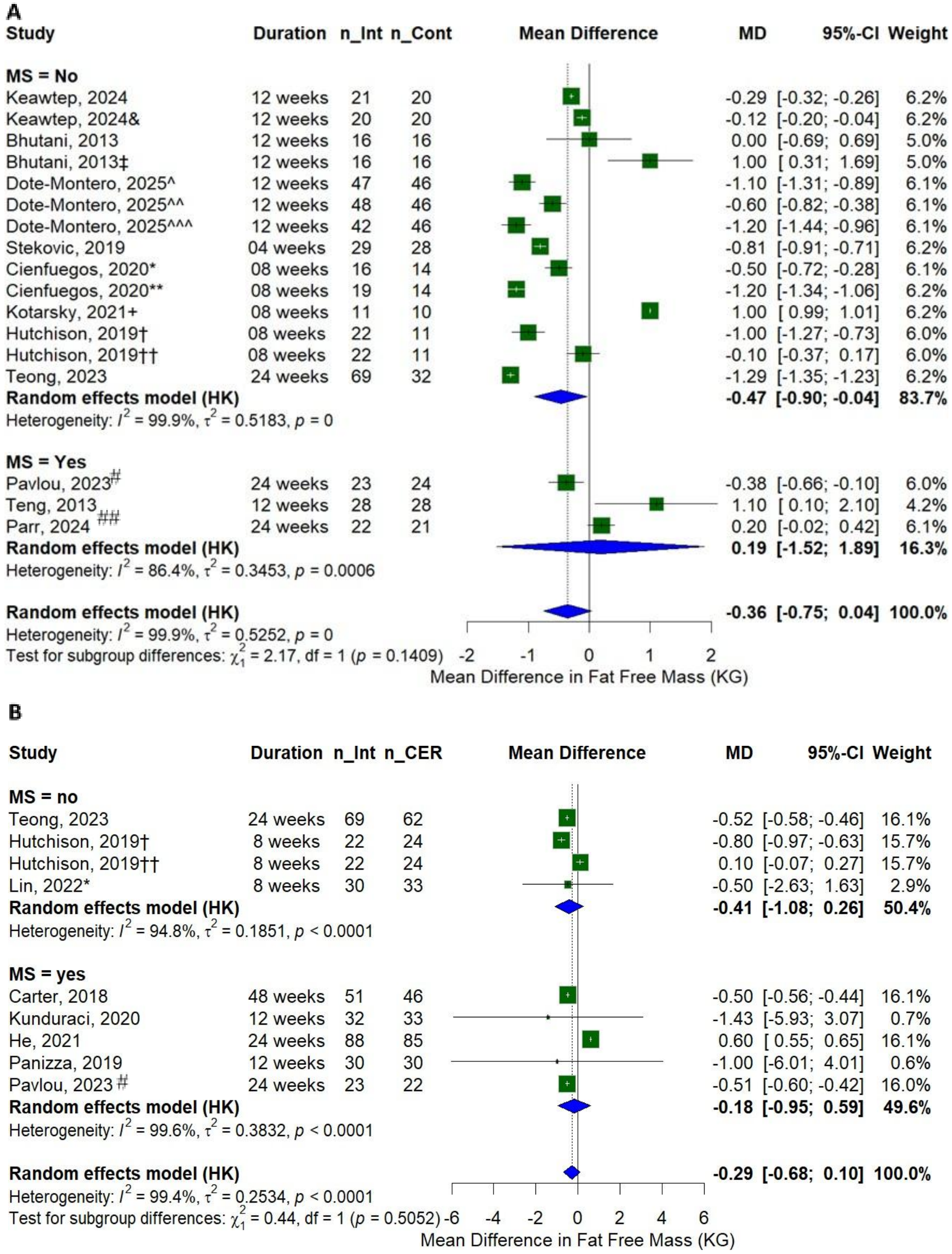

Mean difference indicates the mean difference of change from fasting intervention *vs*. control group. Each horizontal line represents the 95% confidence interval (CI) for an individual study. The green squares indicate the effect estimates from individual studies, with the square size proportional to the study weight. The blue diamond represents the pooled effect estimate, with its width showing the 95% CI. The blue diamond at the base of the plot demonstrates the pooled effect estimates and confidence intervals from all RCTs included.

Marks: [*]4h TRE; [**]6h TRE; [#]8h TRE; [##]9h TRE; [+]8h TRE+physical activity; [^] 8h eTRE; [^^] 8h iTRE; [^^^]8h ssTRE.

Abbreviations: TRE – time restricted eating; eTRE – early time restricted eating, iTRE – intermediate time restricted eating, ssTRE – self-selected time restricting eating; ADF – alternate day fasting; CI – confidence interval; Cont – control group; Int – intervention group; MD – mean difference; n – number of participants in each arm; MS – metabolic syndrome.

Figure S10. Effect of fasting *vs*. control (A-habitual diet as a control, B-CER as a control) on changes in **fat mass** (in kg), stratified by presence of metabolic syndrome.

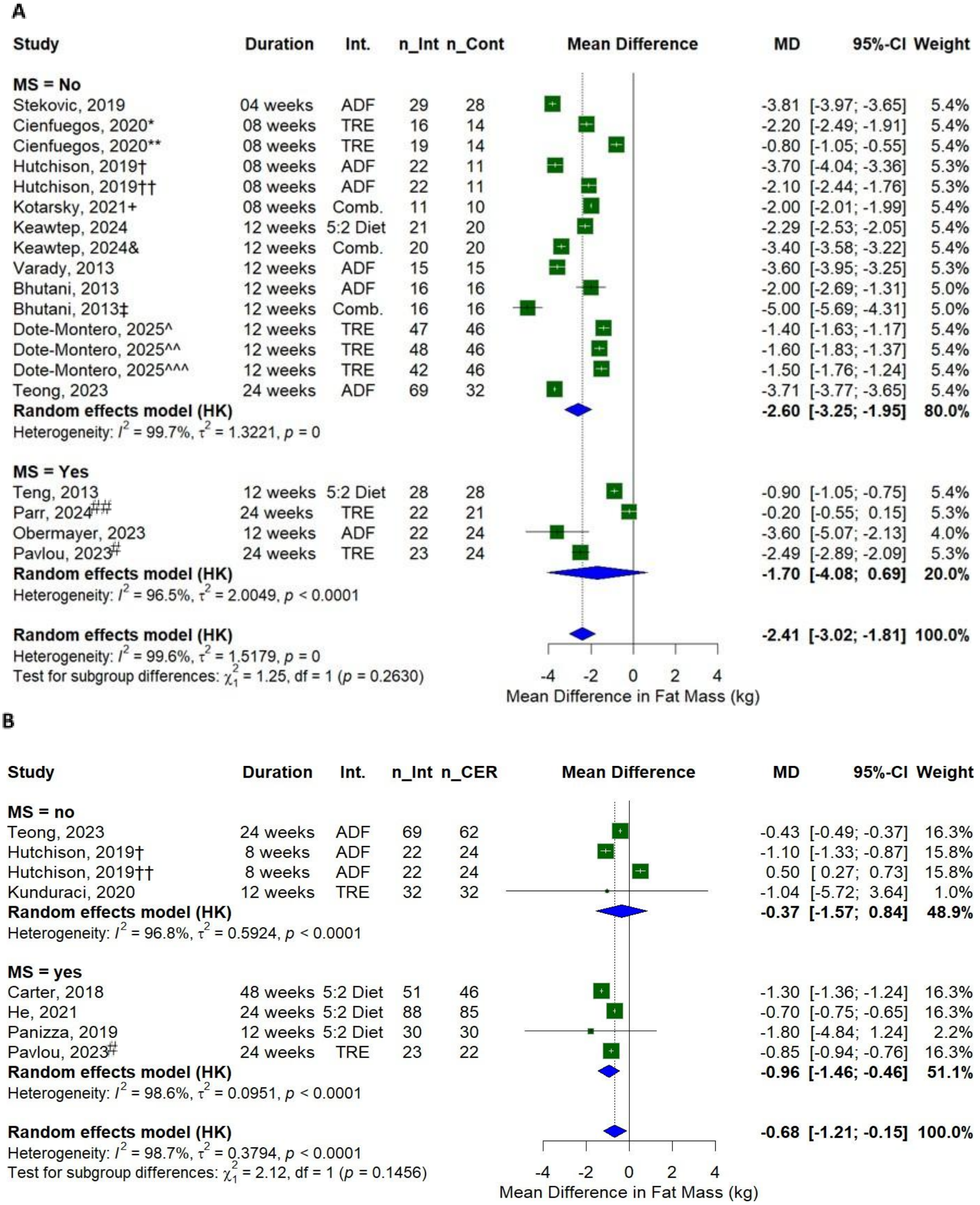

Mean difference indicates the mean difference of change from fasting intervention *vs.* control group. Each horizontal line represents the 95% confidence interval (CI) for an individual study. The green squares indicate the effect estimates from individual studies, with the square size proportional to the study weight. The blue diamond represents the pooled effect estimate, with its width showing the 95% CI. The blue diamond at the base of the plot demonstrates the pooled effect estimates and confidence intervals from all RCTs included.

Marks: *4h TRE; **6h TRE; #8h TRE; ##9h TRE; +8h TRE+physical activity; ^ 8h eTRE; ^^ 8h iTRE; ^^^8h ssTRE.

Abbreviations: TRE – time restricted eating; eTRE – early time restricted eating, iTRE – intermediate time restricted eating, ssTRE – self-selected time restricting eating; ADF – alternate day fasting; CI – confidence interval; Cont – control group; Int – intervention group; MD – mean difference; n – number of participants in each arm; MS – metabolic syndrome.

Figure S11. Effect of fasting *vs*. control (A-habitual diet as a control, B-CER as a control) on changes in **body weight** (in kg), stratified by presence of metabolic syndrome.

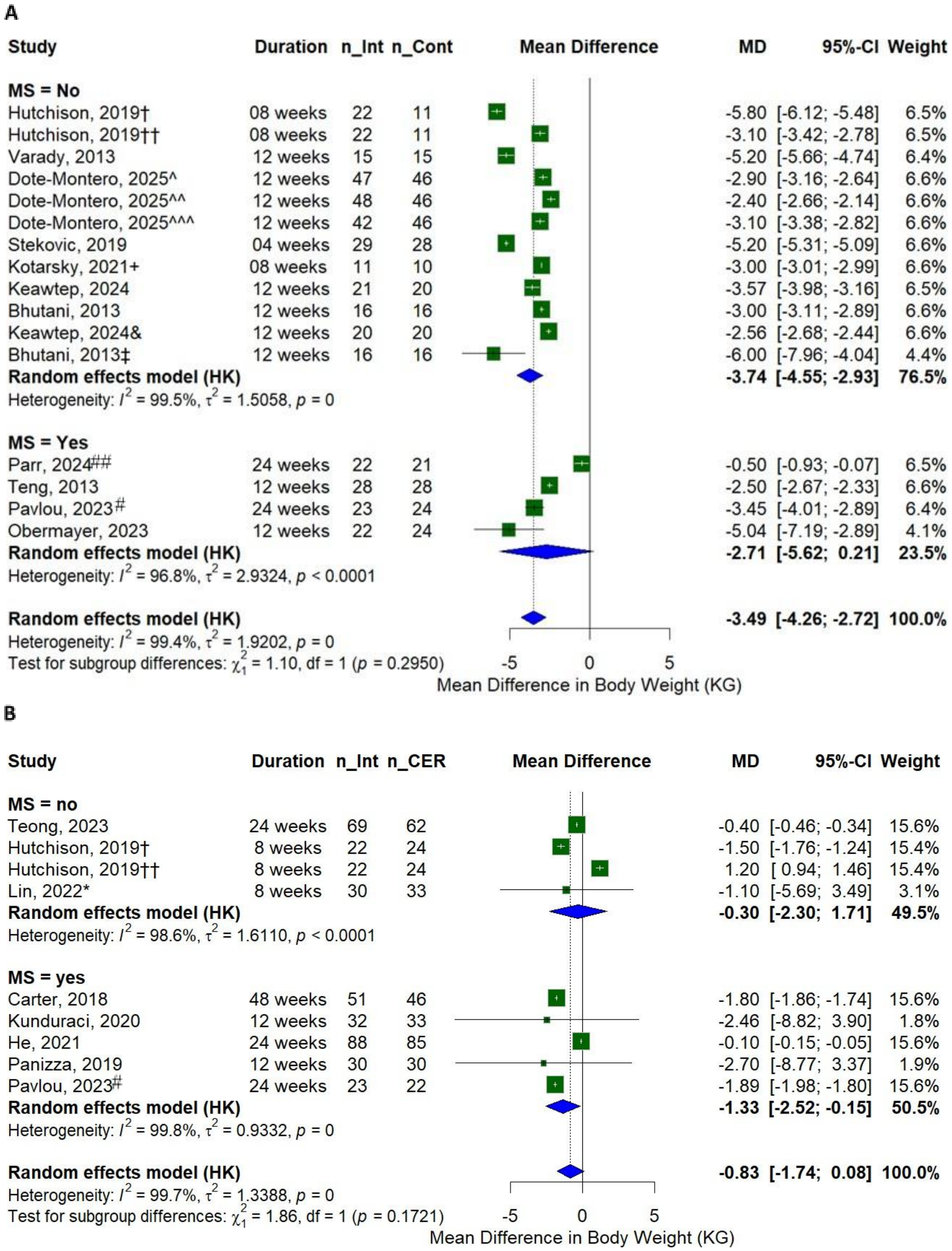

Mean difference indicates the mean difference of change from fasting intervention *vs*. control group. Each horizontal line represents the 95% confidence interval (CI) for an individual study. The green squares indicate the effect estimates from individual studies, with the square size proportional to the study weight. The blue diamond represents the pooled effect estimate, with its width showing the 95% CI. The blue diamond at the base of the plot demonstrates the pooled effect estimates and confidence intervals from all RCTs included.

Marks: [*]4h TRE; [**]6h TRE; [#]8h TRE; [##]9h TRE; [+]8h TRE+physical activity; [^] 8h eTRE; [^^] 8h iTRE; [^^^]8h ssTRE.

Abbreviations: TRE – time restricted eating; eTRE – early time restricted eating, iTRE – intermediate time restricted eating, ssTRE – self-selected time restricting eating; ADF – alternate day fasting; CI – confidence interval; Cont – control group; Int – intervention group; MD – mean difference; n – number of participants in each arm; MS – metabolic syndrome.

Figure S12. Effect of fasting *vs*. control (A-habitual diet as a control, B-CER as a control) on changes in **visceral fat mass** (in kg), stratified by presence of metabolic syndrome.

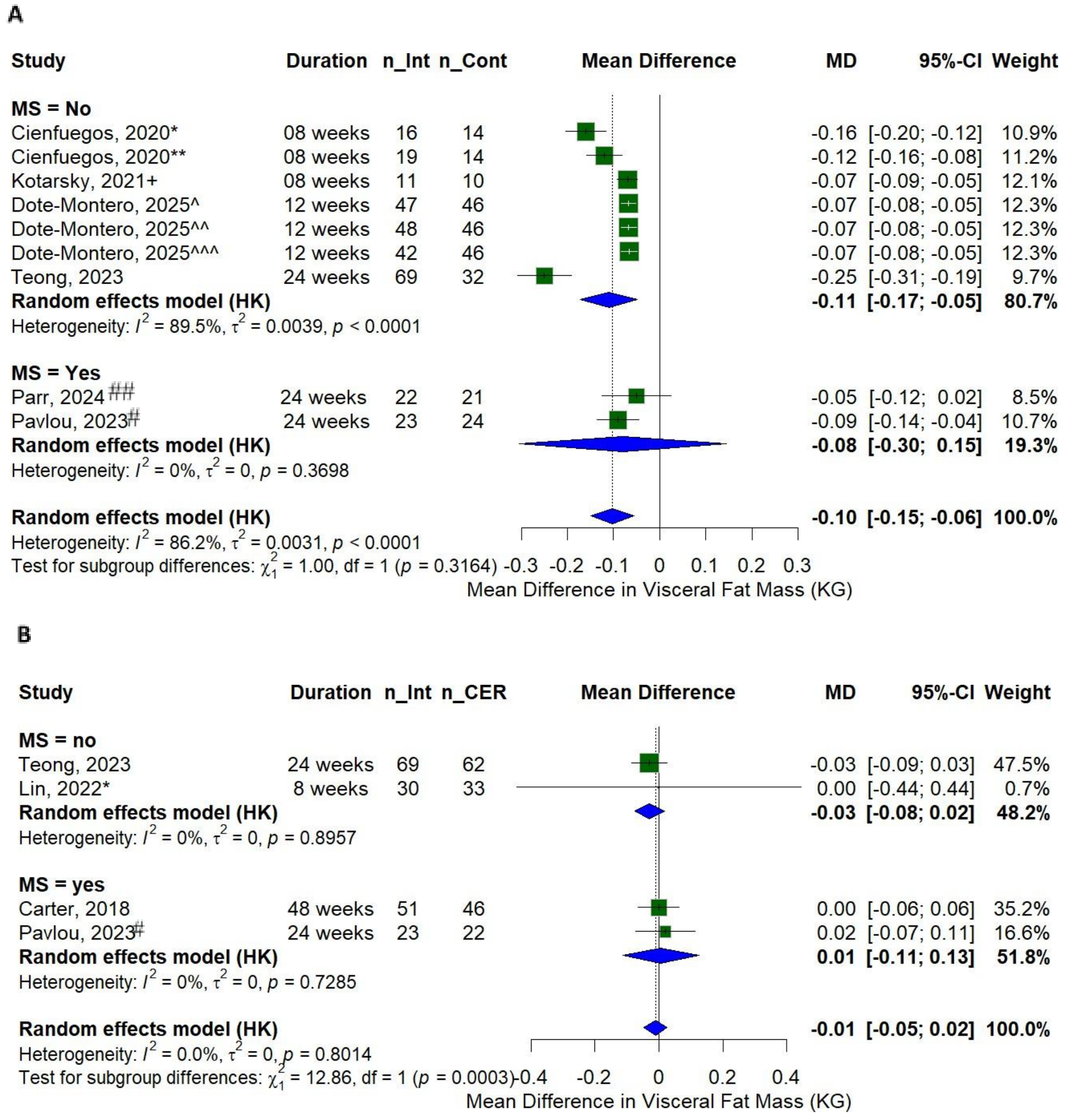

Mean difference indicates the mean difference of change from fasting intervention *vs*. control group. Each horizontal line represents the 95% confidence interval (CI) for an individual study. The green squares indicate the effect estimates from individual studies, with the square size proportional to the study weight. The blue diamond represents the pooled effect estimate, with its width showing the 95% CI. The blue diamond at the base of the plot demonstrates the pooled effect estimates and confidence intervals from all RCTs included.

Marks: *4h TRE; **6h TRE; #8h TRE; ##9h TRE; +8h TRE+physical activity; ^ 8h eTRE; ^^ 8h iTRE; ^^^8h ssTRE.

Abbreviations: TRE – time restricted eating; eTRE – early time restricted eating, iTRE – intermediate time restricted eating, ssTRE – self-selected time restricting eating; ADF – alternate day fasting; CI – confidence interval; Cont – control group; Int – intervention group; MD – mean difference; n – number of participants in each arm; MS – metabolic syndrome.

Figure S13. Effect of fasting *vs*. control (A-habitual diet as a control, B-CER as a control) on changes in **waist circumference** (in cm), stratified by presence of metabolic syndrome.

A

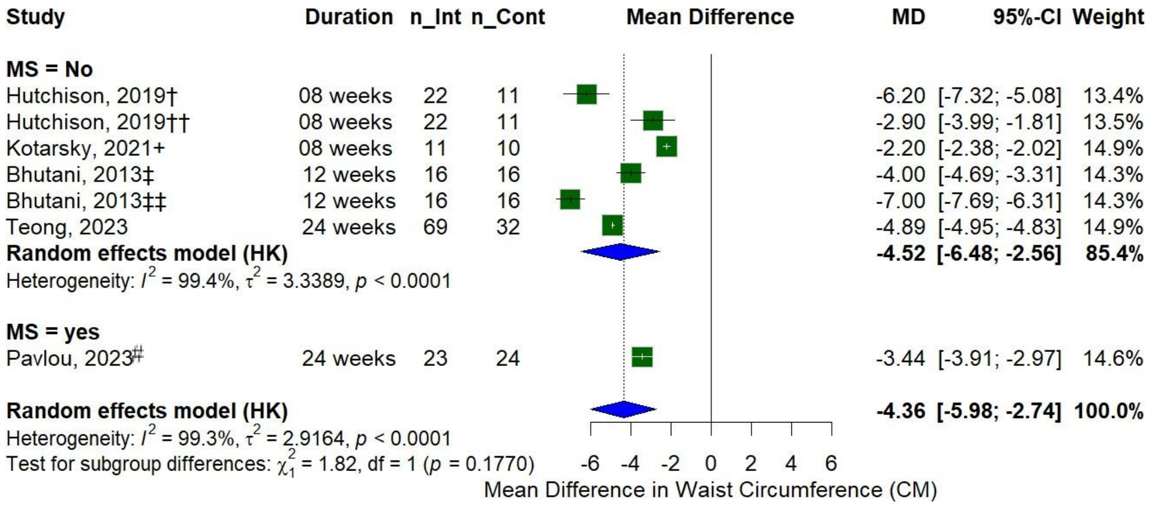

B

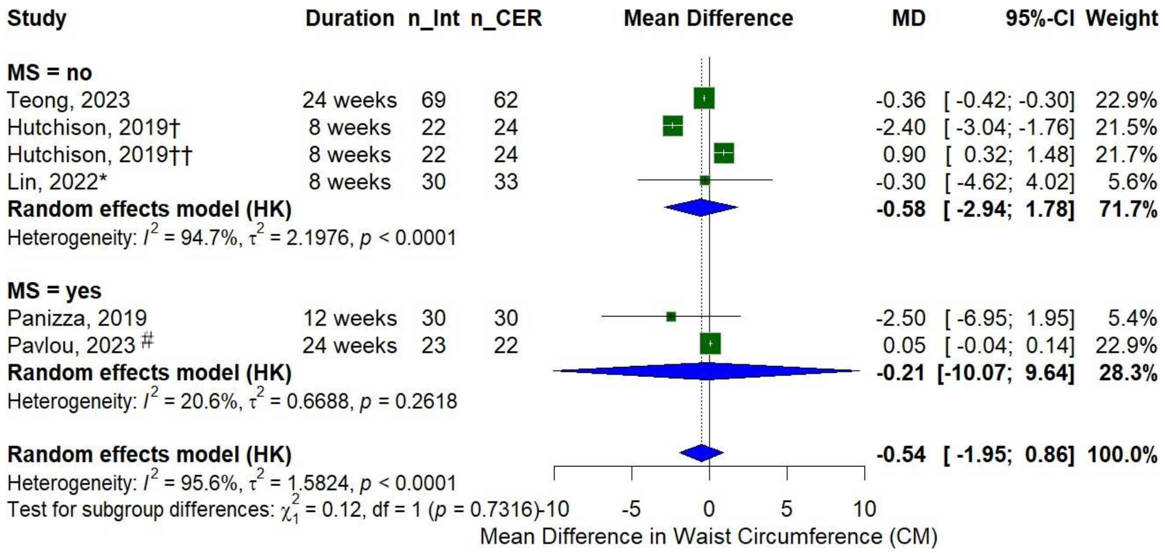

Mean difference indicates the mean difference of change from fasting intervention *vs.* control group. Each horizontal line represents the 95% confidence interval (CI) for an individual study. The green squares indicate the effect estimates from individual studies, with the square size proportional to the study weight. The blue diamond represents the pooled effect estimate, with its width showing the 95% CI. The blue diamond at the base of the plot demonstrates the pooled effect estimates and confidence intervals from all RCTs included.

Marks: †IF70; ††IF100; &5:2 Diet+physical activity; +8h TRE+physical activity; ‡mADF+physical activity; #8h TRE.

Abbreviations: TRE – time restricted eating; ADF – alternate day fasting; IF - intermittent fasting; CI – confidence interval; Cont – control group; Int – intervention group; MD – mean difference; n – number of participants in each arm; MS – metabolic syndrome.
